# A combined field study of Buruli ulcer disease in southeast Benin proposing preventive strategies based on epidemiological, geographic, behavioural and environmental analyses

**DOI:** 10.1371/journal.pgph.0000095

**Published:** 2022-01-07

**Authors:** Alexandra Boccarossa, Horace Degnonvi, Télesphore Yao Brou, Marie Robbe-Saule, Lucille Esnault, Yan Boucaud, Matthieu Eveillard, Ronald Gnimavo, Saturnin Hounsou, Armel Djenontin, Christian Roch Johnson, Sébastien Fleuret, Estelle Marion

**Affiliations:** 1 Univ Angers, Inserm, CRCINA, Angers, France; 2 Univ Angers, CNRS, ESO, Angers, France; 3 University Abomey Calavi, Cifred, Benin; 4 Geography Department, University of La Reunion, Saint-Denis, France; 5 Univ Angers, Inserm, CHU Angers, CRCINA, Angers, France; 6 CDTLUB, Fondation Raoul Follereau, Pobe, Benin; 7 Faculté des Sciences et Techniques, University of Abomey-Calavi, Abomey Calavi, Benin; Rajarata University of Sri Lanka, SRI LANKA

## Abstract

Buruli ulcer is a neglected tropical disease caused by *M*. *ulcerans*, an environmental mycobacterium. This cutaneous infectious disease affects populations with poor access to sanitation, safe water and healthcare living in rural areas of West and Central Africa. Stagnant open bodies of surface water and slow-running streams are the only risk factor identified in Africa, and there is no human-to-human transmission. Appropriate and effective prevention strategies are required for populations living in endemic areas. Based on a multidisciplinary approach in an area in which Buruli ulcer is endemic in South Benin, we investigated the link between all human-environment interactions relating to unprotected water and behaviors associated with Buruli ulcer risk likely to affect incidence rates. We characterised the sources of water as well as water bodies and streams used by communities, by conducting a prospective case-control study directly coupled with geographic field observations, spatial analysis, and the detection of *M*. *ulcerans* in the environment. A full list of the free surface waters used for domestic activities was generated for a set of 34 villages, and several types of human behaviour associated with a higher risk of transmission were identified: (i) prolonged walking in water to reach cultivated fields, (ii) collecting water, (iii) and swimming. Combining the results of the different analyses identified the risk factor most strongly associated with Buruli ulcer was the frequency of contact with unprotected and natural water, particularly in regularly flooded or irrigated lowlands. We confirm that the use of clean water from drilled wells confers protection against Buruli ulcer. These specific and refined results provide a broader scope for the design of an appropriate preventive strategy including certain practices or infrastructures observed during our field investigations. This strategy could be improved by the addition of knowledge about irrigation practices and agricultural work in low-lying areas.

## Introduction

Buruli ulcer is the third most common mycobacterial disease after tuberculosis and leprosy. This infection, caused by an environmental mycobacterium, *Mycobacterium ulcerans*, is characterised by extensive lesions of the skin and soft tissues, potentially resulting in scarring and serious functional disability [1]. This neglected tropical disease may, in rare cases, be fatal, and has a major impact in terms of the stigmatisation, exclusion and poverty of those affected (25% of sufferers are disabled). More than half of all new cases of Buruli ulcer reported occur in countries along the Gulf of Guinea in West Africa: Benin, Cote d’Ivoire, Ghana, Guinea, Liberia, Nigeria, Sierra Leone and Togo [2]. The cases are distributed between well-demarcated geographic areas. The rural zones of West and Central Africa, in which access to water treatment infrastructures and clean drinking water is limited or absent, are the regions most affected by this disease today [3–6]. The rural areas in which this disease is endemic are also generally located close to rivers and low-lying wet plains that are either floodable or irrigated, encompassed by the generic term “lowlands” [7–10].

Buruli ulcer primarily affects children between the ages of 5 and 15 years, with this group accounting for about 50% of new cases in endemic areas in Africa [11]. In some epidemiological studies, women have been reported to be more affected by the infection than men [12], but findings vary between countries [13–15]. No human-to-human transmission of this infection has ever been reported. The ecological characteristics and mode of transmission of *M*. *ulcerans* are not entirely understood, and several fundamental questions remain unanswered [16, 17]. One key concern relates to the routes by which *M*. *ulcerans* crosses the human skin barrier. In West Africa, the emergence and distribution of Buruli ulcer cases are clearly linked to aquatic ecosystems [4, 5, 18–20]. Then, different types of sources of water as well as water bodies and streams were suspected to be a reservoir of *M*. *ulcerans* and many field studies detected the presence *of M*. *ulcerans* DNA in various elements of aquatic food webs in several African countries [9, 21–29]. Ecological and epidemiological studies propose different modes of transmission from aquatic environments, including the passive or active trans-inoculation of *M*. *ulcerans* by plants or aquatic biting insects through human skin [2, 22, 24, 30–32]. Probably due to the mode of penetration of the bacillus into the body, the lesions are often localised on the lower limbs (60% of cases), and on the legs in particular [9]. In this context, the principal risk factor for exposure to *M*. *ulcerans* identified to date in Africa is direct contact, of variable duration, with a slow-flowing or stagnant water source harbouring various aquatic organisms [1, 26, 33]. However, a contamination outside the aquatic environment is not excluded but remains marginal [34–36].

Between the 1980s and 2006, the number of new cases of Buruli ulcer (between 5,000 and 10,000 cases per year according to the WHO) and the number of humid tropical regions affected increased steadily [2, 9]. However, over the last decade or so, we have observed a gradual, unexplained decrease in incidence in West and Central Africa and, conversely, an increase in the number of cases in Australia [37]. Early diagnosis combined with antibiotic treatment and appropriate wound care prevent permanent lesions requiring amputations or resulting in disabling retractions of one or several limbs [38, 39]. However, patient management is often difficult in the zones in which this disease is endemic and rife, particularly in the rural zones of West and Central Africa. It is, therefore, important to develop prevention strategies adapted to the lifestyles of the local communities living in these zones. The formulation of an appropriate and effective prevention strategy requires the precise identification of (i) the conditions rendering an aquatic ecosystem favorable to *M*. *ulcerans*, and (ii) the water-related behaviours and practices that act as risk factors.

The districts of Ouémé and Plateau are among the most endemic for Buruli ulcer in Benin [3]. Our research team has a long-standing partnership with the principal Buruli ulcer care centre, the CDTLUB in Pobé, which is the reference centre for Buruli ulcer management for these two districts [12, 23, 38, 40, 41]. Through this partnership, we were able to assess the differences in Buruli ulcer incidence in the Ouémé and Plateau districts [3]. We then investigated the reasons for this variation, by observing and characterizing the sources of water as well as water bodies and streams used by communities through a prospective case-control study directly coupled with geographic field observations, spatial analysis, and the detection of *M*. *ulcerans* in the environment. Our unique consortium, combining experts in several disciplines (medicine, epidemiology, microbiology, spatial analysis, and health geography), aimed to determine the principal characteristics of the specific unprotected water used by local population in this region of Benin and the factors associated with a risk of *M*. *ulcerans* contamination or protection against such infection. This study was designed to identify the behaviours and territorial practices most strongly linked to *M*. *ulcerans* and to highlight possible modes of community management at the local village scale.

## Materials and methods

We used several methodological approaches: the definition and observation of sources of water as well as water bodies and streams used by the communities, field observations, case-control studies, spatial analysis, and the analysis of environmental samples.

### 1. Definition of sources of water as well as water bodies and streams used by the communities

**Fig 1:**
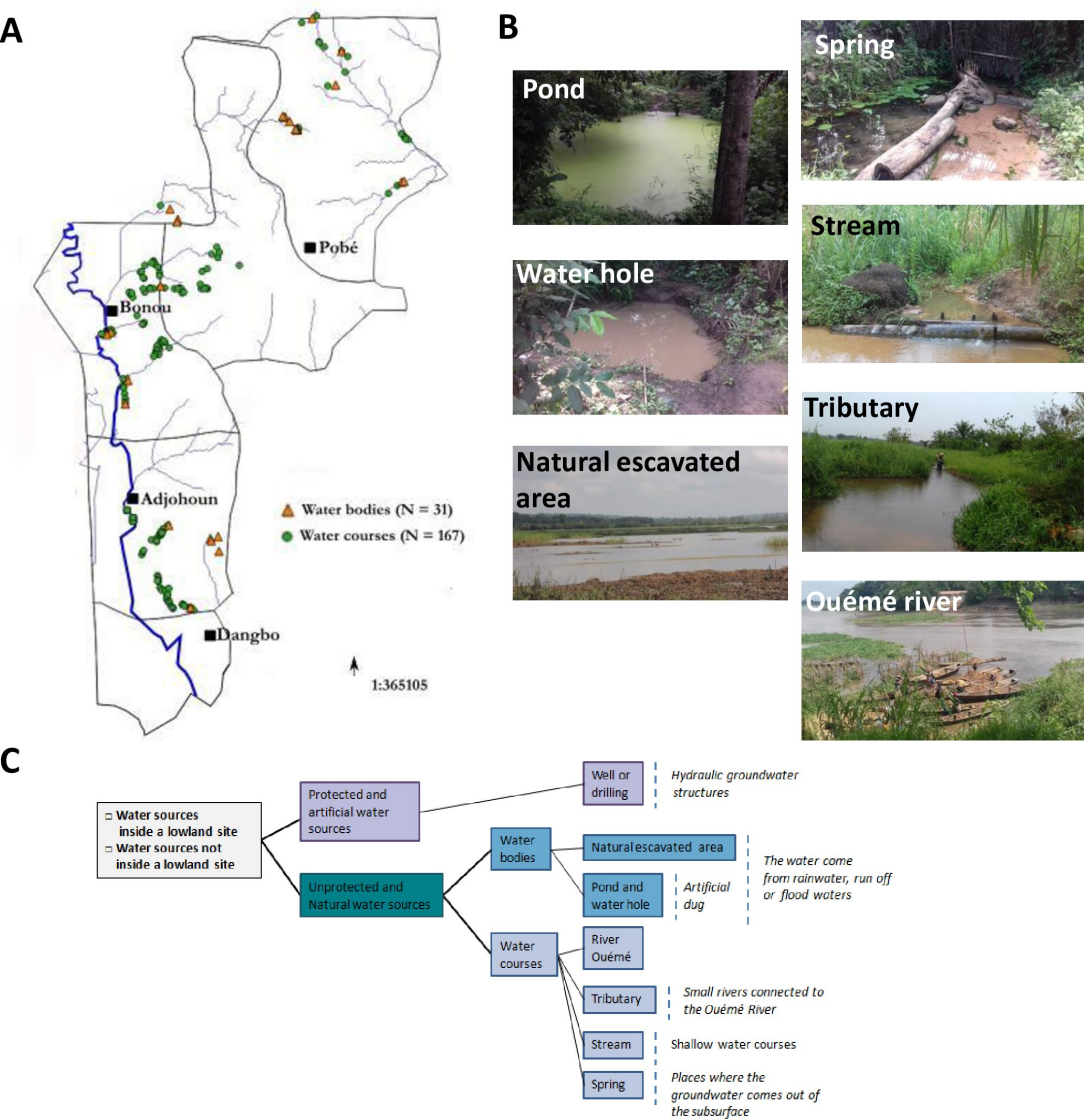
Localisation and diversity of natural water sites used by local population. (A) Localisation of all the unprotected and natural water sites visited in the study area. Datas for the administrative boundary were provided by Ministry of Territory Planning of Benin (https://gadm.org/download_country_v3.html). The map of rivers was provided by Ministry of Mines, Energy and Hydraulics (http://www.hydrosciences.fr/sierem/produits/gis/Oueme.asp). (B) Examples of unprotected water sources. (C) Classification of water sites by type.

We performed a field investigation based on direct observation [42] in 34 villages to compile an exhaustive inventory and specific description of all sources of water as well as water bodies and streams frequently visited by the population (S1 Table and Fig 1). Photographs were taken and global positioning system (GPS) co-ordinates were recorded for 189 identified sources of water as well as water bodies and streams. Another independent characteristic of these sites that was recorded was their presence within “lowland sites” or elsewhere. Due to the proximity of the Ouémé river and its tributaries, “lowland sites” were defined as damp, gently sloping areas (altitudes of 0 to 20 metres above sea level) with a seasonal presence of water due to river flooding and rainfall, or areas flooded by overflowing watercourses at higher altitudes. Sources of water as well as water bodies and streams that never displayed seasonal or intermittent flooding were considered to lie outside lowland sites.

### 2. Field observations

**Fig 2:**
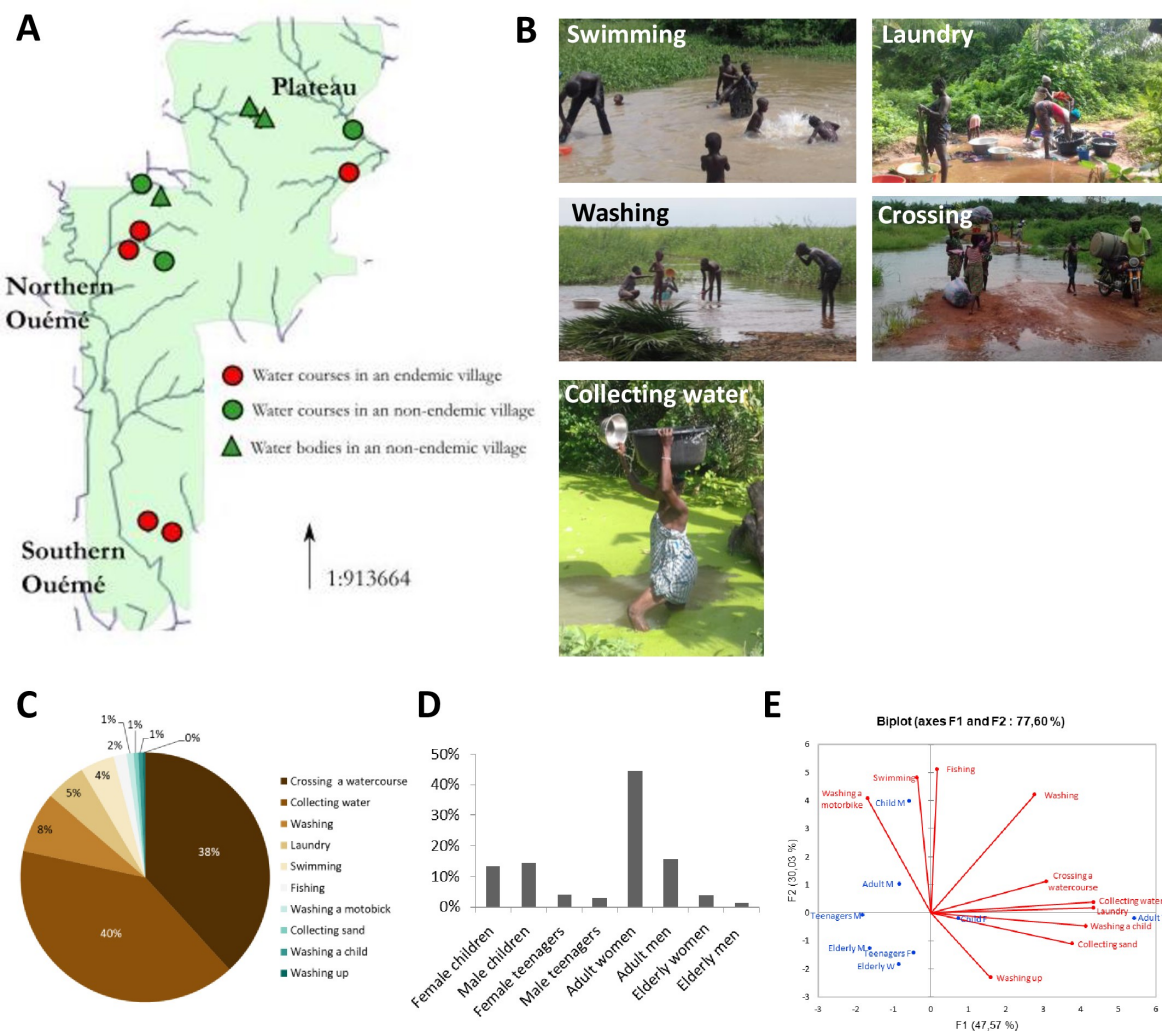
Identification of human activities at natural water sites. (A) Location of the 11 prospected water sites. Datas for the administrative boundary were provided by Ministry of Territory Planning of Benin (https://gadm.org/download_country_v3.html). The map of rivers was provided by Ministry of Mines, Energy and Hydraulics (http://www.hydrosciences.fr/sierem/produits/gis/Oueme.asp). (B) Illustrations of human activities related to unprotected water. (C) Frequency of activities observed at the water sites. (D) Age and sex distribution of people observed at water sites. (E) PCA of water-related activities, age and sex distribution.

We studied the water-related behaviours and activities of the communities through thorough investigations of nine villages, corresponding to 11 sources of water as well as water bodies and streams of different types, over a period of three months (Fig 2A). The various types of individual behaviour were described on the basis of static observations [43, 44]. A four-step method was used: (i) selection of the sources of water as well as water bodies and streams in and around the village, for long-term observations; (ii) initial contact with the group of villages studied at the investigation site, without the collection of information, to enable the communities to identify the researchers, and to ensure the acceptability of subsequent visits (the aim being to observe unobtrusively, so as not to affect usual behaviour); (iii) observation of all activities in and around sources of water as well as water bodies and streams at different times of the day; (iv) quantitative data collection (age and sex, main activities and frequency of visits), recorded on scoring grids, and qualitative data collection based on notes describing the general appearance of the site, and individual and collective behaviours in the aquatic environment. We performed 40 observation sessions (each session lasted approximately three hours) and 1,411 different people were observed during their domestic and recreational water-related activities. Four age groups were defined, for investigations of the links of age and sex to specific activities with respect to sources of water as well as water bodies and streams: 1) Children <15 years old, 2) Teenagers, from 15 to 18 years of age, 3) Adults aged from 19 to 50 years, and 4) Seniors, adults >50 years old.

### 3. Case-control study

#### 3.1 Time frame and study design

We performed a case control study (with two controls matched to each case for the criteria listed below) from January 2018 to December 2020. Prospective epidemiological data were collected from all the communities in which Buruli ulcer is endemic in Ouémé and Plateau, which were covered by the CDTLUB-Pobè during the study period.

#### 3.2 Participants

*Case*. Buruli ulcer cases were defined on the basis of the WHO clinical definition, with confirmation by PCR. Buruli ulcer cases for the case-control study were defined as any patient diagnosed with Buruli ulcer, treated at the CDTLUB-Pobè, and living in the districts of Ouémé and Plateau at the time of diagnosis, between January 2018 and December 2020. Patients who did not live for at least one year in Oueme and Plateau department were not included in the study to avoid the inclusion of patients which were not contaminated in the study area.

*Controls*. Two controls were selected from family members, friends, and neighbors, with matching for age (± 1 year), sex, area of residence, and work occupancy, if possible.

*Procedure for control recruitment*. A door-to-door systematic recruitment was used for the procedure for selecting controls. For any given case, the house of the case was the starting point for control recruitment. We then continued in a random direction from this house if necessary to recruit additional controls. The random walking procedure used here was adapted from the methods detailed in a previous WHO survey [45], which are easy to implement during field investigations in rural areas. We then visited the nearest house along the random trajectory and listed all members of the household, to identify potential controls fulfilling the matching criteria. If several suitable individuals were identified, we selected the person most closely matching the criteria for the case concerned. The procedure was repeated until two suitable controls were identified for each case. Controls were examined to rule out the possibility of an active or healed Buruli ulcer.

#### 3.3 Data collection

A pretested questionnaire was then completed, by reading a pretested questionnaire aloud to the selected participants and them completing it with their answers within their sight. The questionnaire was written in French, and was translated into local languages when read aloud to participants. The patients were interviewed just after their diagnosis, and the corresponding controls were interviewed at about the same time. For most of the youngest participants, family members were present during the interview and confirmed the responses given by the child. The questions related to contact with water and changes in behaviour over time. The interviews were designed to identify the targeted periods and to provide benchmarks over time to minimise bias. The questionnaire is available in the S1 and S2 Datas.

#### 3.4 Variables

The variables recorded in this study include: sociodemographic status (e.g., age, sex, education, region of residence, professional activities), life habits, water uses and activities involving contact with water (e.g., washing clothes, bathing, swimming, drinking, or cooking with river water or well water) and relation to disease (i.e. what did the cases know about Buruli ulcer before they contracted the disease and what did the corresponding controls known during the same period, and any special precautions taken in terms of hygiene, sanitation and other ancestral practices against Buruli ulcer).

#### 3.5 Statistical analysis

Data were processed and analyzed with XLSTAT version 2021.1.1 and Stata/SE version 11.0.

Quality control was performed systematically for all the data collected. Buruli ulcer diagnosis was treated as the dependent variable, and sociodemographic status (e.g., age, sex, education, region of residence, professional activities), life habits, water uses and activities involving contact with water (e.g., washing clothes, bathing, swimming, drinking, or cooking with river water or well water) and relation to disease were considered as independent variables. The dependent variable was dichotomised, and a descriptive analysis was performed for the independent variables. All variables are expressed as proportions.

*Univariate analysis*. For the purposes of univariate and multivariable analysis, independent variables with more than two categories (ethnic group, religion, main professional activities, domestic activities, hunting, fishing, and kitchen gardening) were transformed into dichotomous variables.

*Multivariate analysis*. We also performed a multivariable analysis. The univariate odds ratios (ORs) and 95% CIs were estimated for each variable with a conditional logistic regression model (to account for the matched design) and the significance of differences was assessed in a Wald test. Interactions were tested by introducing interaction terms into the model. Variables with *p*<0.20 in univariate analysis were entered into a multivariate conditional logistic regression model for the simultaneous evaluation of their independent effects. The final model was obtained by a stepwise deletion of variables until *p*<0.05 for all the remaining predictors.

#### 3.6 Consent and ethics committee approval

All the participants included, or their parents or guardians (for individuals under the age of 18 years), received information about the aims of the study. Given the high illiteracy rates among the rural population of the Ouémé and Plateau districts, informed consent was first obtained orally from adults (cases and controls) or from the parents (or legal guardians) of minors (cases and controls), in the presence of the health worker (nurse) as a witness, before enrolment and interview. Then, a written consent was obtained from all participants. We also obtained authorisation for data collection from the medical officers and heads of all the districts in Ouémé and Plateau. Data collection was approved by the institutional review board of the CDTLUB, the national Beninese Buruli ulcer control authorities (IRB00006860), and the National Ethics Committee for Health Research (CNERS) of Benin.

### 4. Spatial analysis

Altitude data were collected from Landsat’s 30-metre SRTM (*Shuttle Radar Topography Mission*). These data were particularly useful for defining the lowlands. Data concerning the area covered by water were extracted from radar images. The Sentinel 1 sensors of the European Space Agency (Copernicus) provided at least one image per month. On the radar images obtained, the pixels corresponding to water have values below 0.05. A monthly time series of Sentinel 1 radar images consisting of 30 images was obtained over the southern part of the Ouémé catchment area for 2018. Using this time series, we were able to extract the area of the Ouémé basin covered by water and to reconstitute, for each district, the area under water for each month of 2018. We also distinguished between areas permanently and seasonally under water. In addition to the radar images, we also used Sentinel 2 optical images and Spot 6 images to map the areas of vegetation seasonally flooded, by calculating the SWI (surface waterproofing index) and NDVI (normalised difference vegetation index). All the maps in this study were produced with the ENVI and QGIS software suites. Principal Component Analysis (PCA) was run using XLSTAT.

### 5. Environmental study

The environmental study was authorised by the Ministry of Sustainable Development under agreement number 011/MCVDD/PF-APA/SA. Sampling procedures, including those for vertebrates, were specifically approved by this committee in the absence of a specific committee dealing with the ethics of animal experimentation.

#### 5.1 Sample collection

When visiting the home place of each patient for the case-control study, the primary and/or secondary unprotected water were identified and sampling was performed within the following days. The 92 sampling sites were spread throughout the Ouémé and Plateau municipalities. The same methods of aquatic sampling were used at each site. Invertebrates and fish were captured with the sweep of a square net, from the surface down to a depth of 0.2 to 1 m, over a distance of 1 m. A sample was considered to correspond to all the insects collected in 10 such sweeps with the net. All insects were preserved in 70% ethanol for laboratory identification. For the detection of *M*. *ulcerans* DNA, the insects were sorted into pooled groups, each including no more than 20 specimens from the same family. For each body of water, we collected plant samples from the predominant living plant species and the second most frequent plant species. Each of these plant samples consisted of one to five plant leaves, stems, or roots, depending on the size of the plant sample. They were placed directly in a clean 100 mL bottle containing 70% ethanol.

#### 5.2 DNA extraction and purification

Samples were transferred to bead beating tubes (3 mm steel beads) containing 3 mL of PBS and were shaken with TissueLyser (Qiagen) for 10 min at 30 Hz. The samples were then transferred to a 50 mL tube and centrifuged at 420 x *g* for 1 min to remove host cell debris. The supernatant was transferred to another 50 mL tube and centrifuged at 4,600 x *g* for 30 min to pellet bacteria. The supernatant was discarded and the pellet was suspended in 750 μL lysis buffer, transferred to a bead beating tube (ZymoBIOMICS DNA Miniprep Kit, Zymo Research) and shaken with TissueLyser (Qiagen) for 10 min at 30 Hz to lyse the bacteria. DNA was extracted according to the kit manufacturer’s protocol and eluted in 50 μL RNase- and DNase-free water.

#### 5.3 qPCR

Oligonucleotide primer and TaqMan probe sequences were selected from the GenBank IS*2404* sequences and the ketoreductase B (KR) domain of the mycolactone polyketide synthase (*mls*) gene from the plasmid pMUM001 [22]. We performed qPCR in a reaction volume of 20 μL containing qPCR mix, 300 nM primers, 100 nM TaqMan probe and 5 μL DNA or sterile water as a negative control. Reactions were run on an AriaMx Thermocycler (Agilent), with the following program: 10 min at 95°C and 40 cycles of 15 s at 95°C and 1 min at 60°C. Quantitative readout assays were set up, based on an external standard curve generated with a ten-fold dilution series (over six orders of magnitude) of *M*. *ulcerans* DNA. Samples were considered positive only if both the IS*2404* sequence and the sequence encoding the KR domain of *mls* were detected, with threshold cycle (Ct) values strictly <36 cycles.

## Results

### 1. Field observation of sources of water as well as water bodies and streams

#### 1.1 Diversity of unprotected water sources used by local population

The results presented here were obtained in a region endemic for Buruli ulcer in southern Benin, including five municipalities located in the Ouémé and Plateau districts (Bonou, Adjohoun, Dangbo in Ouémé and Adja-Ouèrè and Pobè in Plateau). The Ouémé river crosses the three municipalities of the Ouémé district, whereas its tributaries are present in both districts (Fig 1A). There are 210 registered villages (with various incidence rates for Buruli ulcer) in this area, and the epidemiological data for Buruli ulcer were obtained from the main Buruli ulcer treatment centre in Benin (CDTLUB-Pobè). Based on our knowledge of this endemic area [3, 12, 40], we decided to conduct typology work in 34 villages distributed throughout the study area, to assess the diversity of the unprotected and natural sources of water as well as water bodies and streams used by local population (S1 Table). Seven of the villages were considered to be highly endemic for Buruli ulcer (1 to 5 cases per year), 18 were considered endemic (0.2 to 1 cases per year), and nine were considered to be slightly endemic or not endemic for Buruli ulcer (<0.2 per year). We identified 189 natural sources of water as well as water bodies and streams used by the population of the 34 villages for various water-related domestic and leisure activities. Natural sources of water as well as water bodies and streams can be classified into two groups: open water bodies (*n* = 30, 16%), including natural excavated areas (4%) and ponds or water holes (12%) of various shapes and sizes, and water courses (*n* = 159, 84%), including the Ouémé river (7%), its tributaries (21%), streams (24%), and springs (32%) (Fig 1A–1C and S1 Table). Overall, 64% (*n* = 121) of the sources of water as well as water bodies and streams observed were located in lowlands.

This initial typology revealed that the population of Ouémé had a greater diversity of unprotected natural sources as well as water bodies than those living in Plateau, due to the more extensive hydrological network present (S1 Table and Fig 1A). Furthermore, the variables “village endemicity rate” and “number of sources of water as well as water bodies and streams” used by the local population were significantly associated (*p* <0.001). Indeed, the local population of the villages of Ouémé with the highest endemicity rates (from 1 to 5 cases per year) used a mean of nine different sources of water as well as water bodies and streams, whereas those with low or no endemicity for Buruli ulcer used only a mean of five sources of water as well as water bodies and streams. The nature of the sources of water as well as water bodies and streams used was not associated with the risk of contamination. However, endemicity rate was associated with the location of a source of water or water bodies and streams in a lowland area (S1 Table). On one side of this district, there are shallowly sloping lowlands situated along or close to a large plain regularly flooded by the Ouémé river in spate. On the other side, there are low-lying lands that are more circumscribed in space and characterised by shorter periods of flooding due to the accumulation of runoff water or the backing up of a downstream river or stream. For almost 40% of the villages investigated in Ouémé, more than half (in some cases all) the sources/bodies of water used were located at lowland sites, whereas this was the case for only 12% of the villages in Plateau, which also had lower incidence rates.

#### 1.2 Identification of human activities at sources of water as well as water bodies and streams

We thoroughly investigated 11 unprotected natural sources of water as well as water bodies and streams, to identify the types of behaviour and territorial practices most strongly linked to exposure to *M*. *ulcerans* (Fig 2A and 2B). We listed 10 water-related activities (Fig 2C), mainly performed by female adults (45%), male adults (16%), and children aged 1–15 years (13% girls, 14% boys) (Fig 2D). The two most frequent activities were “crossing a water course” (40%) and “collecting water” (38%) (Fig 2C). The activity “collecting water” corresponds to the collection and storage of water in plastic basins or buckets, a task performed mostly by adult women (60%) and girls (19%). During this activity, the time spent in the water does not exceed five minutes, and the water comes into contact with the feet and legs, but only rarely the upper body. The activity “crossing a water course” corresponds to crossing a source/body of water on the way to fields or a neighboring village, or walking along a flooded path or the bed of a stream over various distances. The time spent in contact with the water depends on the nature of the sites and the location of fields, with water level varying considerably between the dry season and periods of flooding. This is a repetitive activity performed mostly by adult women (39%).

The other eight activities accounted for about 22% of total activities. Recreational activities were recorded (4.5%), including swimming, mostly by young boys (71%). Young children (<3 years of age) waded in the water close to their mothers, whereas older children tended to swim away from the main points of entry into the water, with their legs and arms in the water. Many swimming sites were observed away from the principal water sources, and these sites were frequented by children without adult supervision. Washing activities occurred at various times of the day. Laundry (5%) was the washing activity involving the longest period of contact with the aquatic environment (71 min on average). This activity takes place in the direction of the flow of water courses and at the cleanest points of water sources access. Washing dishes was only rarely observed (0.30%) because this domestic activity is mostly performed at home.

Principal component analysis (PCA) indicated strong associations between adult women and the activities “crossing a water course” and “collecting water”, but also between this group of individuals and water-related washing activities in the broadest sense (Fig 2E). The PCA also distinguished clearly between adult women and boys, who were specifically linked to swimming (recreational activity) and washing motorbikes.

These global results provide an indication of the types of activities performed and the types of contact between humans and unprotected water. There were, of course, differences between the sources/bodies of water evaluated in our study area. Not all of the 10 activities studied were observed everywhere. For example, in Issaba village (not endemic for Buruli ulcer), the activities performed in ponds are strictly controlled (prohibition of swimming, washing, or entering the water for fishing) to preserve water quality. Water is mostly collected by adult women, whereas boys are involved in fishing, during which they remain on the banks of the ponds without coming into direct contact with the aquatic environment. Conversely, in Eguelou village (endemic for Buruli ulcer), water-related activities are performed by a greater diversity of people (although adult women are the principal group involved in supplying water), mostly in direct contact with the water. Finally, certain village practices or infrastructures have been identified as protective against infection, although they were not initially implemented for this reason. Wooden bridges built by the population of Illemon village (not endemic for Buruli ulcer) make it possible to cross a watercourse without coming into direct contact with the aquatic ecosystems, whereas the use of wooden boats and earthen dikes make it possible to move around the lowland site of Yokon village without systematically entering the water.

### 2. A prospective case-control study

#### 2.1 Description of the study population

**Fig 3:**
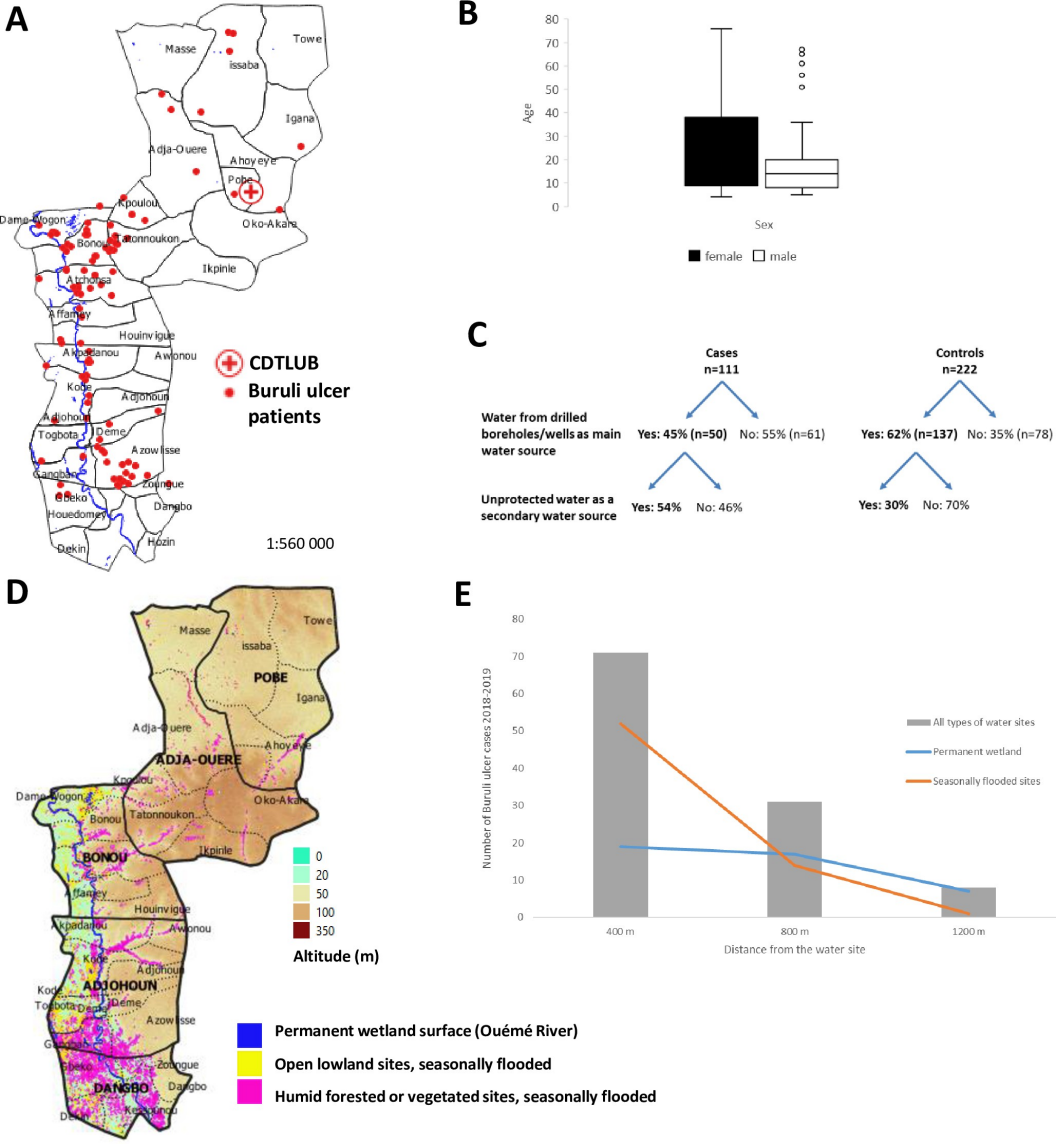
Factors involved in Buruli ulcer disease. (A) Location of Buruli ulcer patients participating in the prospective case-control study. Datas for the administrative boundary were provided by Ministry of Territory Planning of Benin (https://gadm.org/download_country_v3.html). (B) Age and sex distribution of Buruli ulcer patients. (C) Proportion of patients using clean water from drilled boreholes/wells and/or water from unprotected water sites. (D) Mapping of lowlands by spatial analysis. Datas for the altitude and land surface are available on https://earthexplorer.usgs.gov/ (NASA), and https://apps.sentinel-hub.com/eo-browser/ (European Spatial Agency). (E) Number of Buruli ulcer cases according to distance from the river or a lowland water site.

We enrolled 111 cases and 222 controls in the study during the 2018–2020 period. The participants lived in villages in the Ouémé (79.3%) and Plateau (20.7%) districts of Benin (Fig 3A). For cases, median age at diagnosis was 15 years (IQR 9–36 years; mean: 24 years), and a large proportion of the patients were schoolchildren and students (58 [52.3%] patients). The overall sex ratio of the patients was 0.61 at the time of Buruli ulcer diagnosis (*69* [62.2%] female, *42* [37.8%] male, p = 0.02). A distortion of the sex ratio as a function of age was observed, with a strong female preponderance in patients > 15 years (women [71.7%], men [28.3%], *p*< 0.0001) (Fig 3B).

#### 2.2 Sociodemographic and professional characteristics of patients and controls

Each patient was matched with two controls for age, sex, and location of residence, to obtain a case-control ratio of 1:2. Ethnic group and main professional activity were similar between patients and controls (Table 1).

**Table 1:** Sociodemographic, professional, and clinical characteristics of BU cases and controls matched for sex and age (± 1 year), Benin, 2018–20.

Characteristic	Cases (%) (*n* = 111)	Controls (%) (*n* = 222)	Univariate OR	95% CI	*P* value
***Demographic***					
**SEX**					
**M**	42 (37.8)	84 (37.8)	NA	-	NA
**F**	69 (62.2)	138 (62.2)	NA	-	NA
**Age**					
**≤ 15**	58 (52.3)	113 (50.9)	NA	-	NA
**> 15**	53 (47.7)	109 (49.1)	NA	-	NA
**Ethnic group Fon**	10 (9.1)	30 (13.5)	0.56	0.24–1.32	0.186
**Ethnic group Goun**	81 (72.9)	158 (71.2)	1.19	0.59–2.40	0.638
**Ethnic group Holi**	14 (12.6)	26 (11.7)	1.3	0.38–4.45	0.671
**Ethnic group Nago**	6 (5.4)	8 (3.6)	1 (reference)	-	-
***Religion***					
Catholic	29 (26.2)	83 (37.4)	0.5	0.25–0.87	0.017
Celestial Church of Christ	26 (23.4)	34 (15.3)	1.8	0.97–3.27	0.061
Evangelical	47 (42.3)	81 (36.5)	1.4	0.81–2.26	0.255
Muslim	3 (2.7)	10 (4.5)	0.6	0.14–2.19	0.399
Traditional	6 (5.4)	13 (5.9)	0.9	0.26–2.96	0.838
No religion	0 (0)	1 (0.5)	1 (reference)	-	-
***Main professional activities/occupations***					
Craftsman	21 (18.9)	36 (16.2)	1.3	0.64–2.63	0.468
Farmer	13 (11.7)	22 (9.9)	1.5	0.50–4.48	0.467
Trader	20 (18.0)	39 (17.6)	1.1	0.51–2.13	0.903
Schoolchild	49 (44.1)	101 (45.5)	0.8	0.37–1.91	0.672
Public official	1 (0.9)	4 (1.8)	0.4	0.04–4.61	0.489
None	7 (6.3)	20 (9.01)	1 (reference)	-	-
**Clinical form of BU at onset**					
Nodule	60(54.1)	-	-	44.8–63.3	-
Oedema	49 (44.1)	-	-	34.9–53.4	-
Plaque	2 (1.8)	-	-	0.0–4.3	-

**Note:** The data presented are the no. (%) of participants. OR, unadjusted odds ratio for comparison of the case to the two corresponding controls; CI, confidence interval. NA = not applicable because of matching.

#### 2.3 Analysis of general lifestyle characteristics

Univariate analysis showed that the presence of more than 4 people in the household and being the person who systematically went to fetch water (Table 2) were significantly associated with the risk of contracting Buruli ulcer. Conversely, always cooking at home, washing dishes at home, doing the shopping, and using clean water from drilled wells were protective against Buruli ulcer (Table 2).

**Table 2:** Univariate analysis of selected variables for the general lifestyle characteristics of the cohort, Benin, 2018–20.

Characteristics	Cases (%) (*n* = 111)	Controls (%) (*n* = 222)	Univariate OR	95% CI	*P* value
**Number of people/household: ≤ 4 vs. >4**	37 (33.3)	90 (40.5)	1.1	1.02–1.23	0.020
***Responsibility for domestic activities***					
**Cooking**					
Never	49 (44.1)	98 (44.1)	1 (reference)	-	-
Sometimes	34 (30.6)	51 (22.9)	1.6	0.92–2.88	0.098
Always	28 (25.2)	73 (32.9)	0.49	0.25–0.99	0.048
**Washing dishes at home**					
Never	35 (31.5)	70 (31.5)	1 (reference)	-	-
Sometimes	46 (41.4)	66 (29.7)	1.9	1.10–3.15	0.021
Always	30 (27.1)	86 (38.7)	0.42	0.22–0.80	0.008
**Laundry at home**					
Never	33 (29.7)	57(25.7)	1 (reference)	-	-
Sometimes	24 (21.6)	52 (23.4)	0.9	0.47–1.64	0.673
Always	54 (48.7)	113 (50.9)	0.9	0.47–1.56	0.606
**Cleaning the house**					
Never	33 (29.7)	59 (26.6)	1 (reference)	-	-
Sometimes	31 (27.9)	66 (29.7)	0.9	0.50–1.59	0.698
Always	47 (42.4)	97 (43.7)	0.9	0.51–1.64	0.766
**Fetching water**					
Never	33 (29.7)	87 (39.2)	1 (reference)	-	-
Sometimes	25 (22.5)	50 (22.5)	1	0.55–1.81	1
Always	53 (47.8)	85 (38.3)	1.8	1.01–3.13	0.047
**Washing motorcycles/other vehicles**					
Never	101 (90.9)	197 (88.7)	1 (reference)	-	-
Sometimes	6 (5.4)	19 (8.6)	0.6	0.22–1.56	0.285
Always	4 (3.6)	6 (2.7)	1.4	0.34–6.11	0.619
**Shopping**					
Never	54 (48.6)	106 (47.7)	1 (reference)	-	-
Sometimes	31 (27.9)	45 (20.3)	1.6	0.90–2.69	0.110
Always	26 (23.5)	71 (32)	0.5	0.25–0.96	0.039
**Hunting**					
Never	94 (84.7)	195 (87.8)	1 (reference)	-	-
Sometimes	16 (14.4)	24 (10.8)	2	0.7–5.6	0.188
Always	1 (0.9)	3 (1.4)	0.7	0.07–6.41	0.725
**Fishing**					
Never	86 (77.5)	187 (84.2)	1 (reference)	-	-
Sometimes	18 (16.2)	29 (13.1)	1.6	0.66–3.66	0.315
Always	7 (6.3)	6 (2.7)	3.4	0.83–13.56	0.088
**Kitchen gardening**					
Never	86 (77.5)	174 (78.4)	1 (reference)	-	-
Sometimes	18 (16.2)	40 (18.1)	0.9	0.46–1.65	0.666
Always	7 (6.3)	8 (3.5)	2.1	0.63–6.81	0.229
**Use of water from a protected source (drilled borehole or well**					
Yes	50 (45.1)	137 (61.7)	0.20	0.09–0.44	0.000
No	61 (54.9)	85 (38.3)	1 (reference)	-	-

**Note:** The data shown are the no. (%) of participants. OR, unadjusted odds ratio comparing each case to the two corresponding controls; CI, confidence interval. NA = not applicable because of matching.

In our multivariable model (Table 3), the following risk factors were independently associated with Buruli ulcer: being the person systematically responsible for fetching water (OR = 3.4; 95% CI = 1.43–8.22) and households with > 4 members (OR = 1.2; 95% CI = 1.04–1.29). By contrast, washing the dishes at home (OR = 0.4; 95% CI = 0.15–0.97), doing the shopping (OR = 0.4; 95% CI = 0.13–0.90), and using water from protected sources (drilled boreholes and wells) (OR = 0.2; 95% CI = 0.08–0.44) were protective against Buruli ulcer (Table 3). We investigated the factor “using water from protected sources (drilled boreholes and wells)” in more detail. The use of a protected principal water source was more frequent in the control group than in the case group (*p-value* = 0.004, Fig 3C). Even when cases used protected water sources as their principal source of water, they were more likely than the controls to use an unprotected water source as a secondary water source (54% vs. 30%; *p-value* = 0.005, Fig 3C).

**Table 3:** Multivariable analysis showing the general lifestyle factors of the cohort independently associated with Buruli ulcer, Benin, 2018–2020.

Variables	Adjusted OR (95% CI)	*p-*value
**Washing dishes at home**		
Never	1 (reference)	
Sometimes	1.1 (0.55–2.34)	0.739
Always	0.4 (0.15–0.97)	0.042
**Shopping**		
Never	1 (reference)	
Sometimes	0.9 (0.44–1.89)	0.798
Always	0.4 (0.13–0.90)	0.030
**Fetching water from outside**		
Never	1 (reference)	
Sometimes	1.6 (0.70–3.50)	0.273
Always	3.4 (1.43–8.22)	0.006
**Use of clean water from a drilled borehole or well**	0.2 (0.08–0.44)	0.000
**Number of people per household**	1.2 (1.04–1.29)	0.008

**Note:** OR, unadjusted odds ratio for the comparison of each case with its two corresponding controls; CI, confidence interval.

#### 2.4 Analysis of behavioural factors and activities bringing humans into contact with water

We analysed the behavioural factors and human activities associated with a risk of *M*. *ulcerans* infection for cases (*n* = 88) and controls (*n* = 176) living along the Ouémé river, using the classification of sources/bodies of water defined in our field observations. Univariate analysis showed that the use of water sources located in regularly flooded or irrigated lowland sites, coming into contact with external sources of water for the purposes of water collection, washing, swimming, doing the laundry or playing several times per day, doing the laundry alone, washing dishes several times per day, entering a body of water to wash dishes, washing dishes alone, and entering a body of water for the purposes of fishing (Table 4) were significantly associated with the risk of contracting Buruli ulcer. By contrast, collecting water outside, wearing sandals or other footwear exposing the feet, and having some knowledge about Buruli ulcer before infection (or at the time at which the questionnaire was completed for controls), awareness of healthcare campaigns concerning the risks of Buruli ulcer, transmission and the use of special precautions based on hygiene, sanitation or ancestral practices were protective against Buruli ulcer (Table 4).

**Table 4:** Univariate analysis of the activities involving contact with water for BU cases and controls matched for sex and age (± 1 year) living along the river Ouémé, Benin, 2018–20.

Characteristics	Cases (%) (*n* = 88)	Controls (%) (*n* = 176)	Univariate OR	95% CI	*P* value
**Main water point**					
Lowland	15 (17.0)	16 (9.1)	13.7	1.70–110.09	0.014
No Lowland	73 (83)	160 (90.9)	-	-	
**River water**					
Yes	24 (27.3)	32 (18.2)	14.7	1.86–117.09	0.011
No	64 (72.7)	144 (81.8)	1 (reference)	-	-
**Stream**					
Yes	13 (14.8)	20 (11.4)	1.8	0.62–5.40	0.278
No	75 (85.2)	156 (88.6)	1 (reference)	-	-
**Source**					
Yes	13 (14.8)	11 (6.25)	8.3	1.78–38.69	0.007
No	75 (85.2)	165 (93.75)	1 (reference)	-	-
**Fetching water from outside occasionally**					
Yes	2 (2.3)	12 (6.8)	0.2	0.026–1.56	0.125
No	86 (97.7)	164 (93.2)	1 (reference)	-	-
**Fetching water from outside once per week**					
Yes	11 (12.5)	10 (5.7)	2.2	0.93–5.18	0.071
No	77 (87.5)	166 (94.3)	1 (reference)	-	-
**Fetching water from outside daily**					
Yes	10 (11.4)	27 (15.3)	0.7	0.31–1.55	0.368
No	78 (88.6)	149 (84.7)	1 (reference)	-	-
**Fetching water from outside several times per day per day**					
Yes	32 (36.4)	45 (25.6)	2	1.03–3.89	0.041
No	56 (63.6)	131 (74.4)	1 (reference)	-	-
**Fetching water from outside by entering a body of water**					
Yes	42 (47.7)	50 (28.4)	4.6	2.06–10.40	0.000
No	46 (52.3)	126 (71.6)	1 (reference)	-	-
**Fetching water from outside in sandals or other non-closed footwear shoes**					
Yes	17 (19.3)	53 (30.1)	0.44	0.21–0.93	0.030
No	71 (80.7)	123 (69.9)	1 (reference)	-	-
**Fetching water from outside**					
Alone	32 (36.4)	58 (32.9)	1.3	0.65–2.43	0.498
Accompanied	56 (63.6)	118 (67.1)	1 (reference)	-	-
**Washing outside occasionally**					
Yes	6 (6.8)	20 (11.4)	0.5	0.19–1.42	0.200
No	82 (93.2)	156 (88,6)	1 (reference)	-	-
**Washing outside once per week**					
Yes	13 (14.8)	18 (10.2)	1.6	0.71–3.78	0.252
No	75 (85.2)	158 (89.8)	1 (reference)	-	-
**Washing outside daily** per day					
Yes	14 (15.9)	20 (11.4)	1.5	0.71–3.33	0.279
No	74 (84.1)	156 (88.6)	1 (reference)	-	-
**Washing outside several times per day**					
Yes	34 (38.6)	37 (21.0)	5.6	2.24–14.03	0.000
No	54 (61.4)	139 (79)	1 (reference)	-	-
**Washing outside by entering a body of water**					
Yes	64 (72.7)	84 (47.7)	4.89	2.31–10.34	0.000
No	24 (27.3)	92 (52.3)	1 (reference)	-	-
**Washing outside wearing sandals or other non-closed footwear**					
Yes	7 (7.9)	28 (15.9)	0.39	0.15–1.02	0.054
No	81 (92.1)	148 (84.1)	1 (reference)	-	-
**Washing outside**					
Alone	28 (31.8)	39 (22.2)	2	0.99–4.02	0.051
Accompanied	60 (68.2)	137 (77.8)	1 (reference)	-	-
**Swimming occasionally**					
Yes	3 (3.4)	8 (4.5)	0.7	0.15–3.09	0.619
No	85 (96.6)	168 (95.5)	1 (reference)	-	-
**Swimming weekly**					
Yes	4 (4.5)	6 (3.4)	1.4	0.36–5.29	0.639
No	84 (95.5)	170 (96.6)	1 (reference)	-	-
**Swimming daily**					
Yes	5 (5.7)	9 (5.1)	1.1	0.36–3.49	0.845
No	83 (94.3)	167 (94.9)	1 (reference)	-	-
**Swimming several times per day**					
Yes	22 (25.0)	21 (11.9)	4.5	1.76–11.73	0.002
No	66 (75.0)	155 (88.1)	1 (reference)	-	-
**Swimming with full immersion of the body**					
Yes	33 (37.5)	44 (25.0)	2.8	1.30–6.11	0.009
No	55 (62.5)	132 (75.0)	1 (reference)	-	-
**Swimming in sandals or other non-closed footwear**					
Yes	3 (3.4)	11 (6.3)	0.5	0.14–1.95	0.334
No	85 (96.6)	165 (93.7)	1 (reference)	-	-
**Swimming**					
Alone	17 (19.3)	22 (12.5)	2.9	0.99–8.52	0.053
Accompanied	71 (80.7)	154 (87.5)	1 (reference)	-	-
**Laundry occasionally**					
Yes	5 (5.7)	12 (6.8)	0.8	0.27–2.46	0.715
No	83 (94.3)	164 (93.2)	1 (reference)	-	-
**Laundry weekly**					
Yes	23 (26.1)	36 (20.5)	1.7	0.77–3.58	0.200
No	65 (73.9)	140 (79.5)	1 (reference)	-	-
**Laundry daily**					
Yes	10 (11.4)	14 (7.9)	1.5	0.63–3.64	0.357
No	78 (88.6)	162 (92.1)	1 (reference)	-	-
**Laundry several times per day**					
Yes	13 (14.8)	10 (5.7)	4.6	1.46–14.76	0.009
No	75 (85.2)	166 (94.3)	1 (reference)	-	-
**Laundry by entering a body of water**					
Yes	24 (27.3)	45 (25.6)	1.1	0.58–2.18	0.734
No	64 (72.7)	131 (74.4)	1 (reference)	-	-
**Laundry whilst wearing sandals or other non-closed footwear**					
Yes	12 (13.6)	31 (17.6)	0.7	0.31–1.54	0.360
No	76 (86.4)	145 (82.4)	1 (reference)	-	-
**Laundry outside**					
Alone	29 (32.9)	33 (18.8)	2.9	1.39–6.10	0.004
Accompanied	59 (67.1)	143 (81.2)	1 (reference)	-	-
**Washing the dishes occasionally**					
Yes	7 (7.9)	5 (2.8)	2.8	0.89–8.82	0.079
No	81 (92.1)	171 (97.2)	1 (reference)	-	-
**Washing the dishes weekly**					
Yes	9 (10.2)	9 (5.1)	2.4	0.82–6.88	0.109
No	79 (89.8)	167 (94.9)	1 (reference)	-	-
**Washing the dishes daily**					
Yes	5 (5.7)	7 (4.0)	1.4	0.45–4.50	0.542
No	83 (94.3)	169 (96.0)	1 (reference)	-	-
**Washing the dishes several times per day**					
Yes	16 (18.2)	14 (7.9)	4.9	1.59–15.49	0.006
No	72 (81.8)	162 (92.1)	1 (reference)	-	-
**Washing dishes by entering a body of water**					
Yes	37 (42.0)	33 (18.7)	5.6	2.53–12.43	0.000
No	51 (58.0)	143 (81.3)	1 (reference)	-	-
**Washing dishes whilst wearing sandals or other non-closed footwear**					
Yes	7 (7.9)	14 (7.9)	1	0.35–2.83	1.000
No	81 (92.1)	162 (92.1)	1 (reference)	-	-
**Washing dishes**					
Alone	23 (26.1)	20 (11.4)	5.5	2.02–15.08	0.001
Accompanied	65 (73.9)	156 (88.6)	1 (reference)	-	-
**Fishing occasionally**					
Yes	3 (3.4)	5 (2.8)	1.3	0.24–6.89	0.773
No	85 (96.6)	171 (97.2)	1 (reference)	-	-
**Fishing weekly**					
Yes	8 (9.1)	7 (4.0)	3.2	0.92–11.00	0.068
No	80 (90.9)	169 (96.0)	1 (reference)	-	-
**Fishing daily**					
Yes	3 (3.4)	4 (2.3)	1.6	0.31–8.40	0.567
No	85 (96.6)	172 (97.7)	1 (reference)	-	-
**Fishing several times per day**					
Yes	3 (3.4)	0 (0.0)	-	-	-
No	85 (96.6)	176 (100)	1 (reference)	-	-
**Fishing by entering a body of water**					
Yes	17 (19.3)	16 (9.1)	6.6	1.83–23.81	0.004
No	71 (80.7)	160 (90.9)	1 (reference)	-	-
**Fishing whilst wearing sandals or other non-closed footwear**					
Yes	1 (1.1)	5 (2.8)	0.3	0.03–3.12	0.311
No	87 (98.9)	171 (97.2)	1 (reference)	-	-
**Fishing**					
Alone	7 (7.9)	7 (3.9)	7.2	0.81–64.17	0.077
Accompanied	81 (92.1)	169 (96.1)	1 (reference)	-	-
**Knowledge about Buruli ulcer**					
Yes	57(64.8)	142(80.7)	0.4	0.22–0.75	0.004
No	31 (35.2)	34(19.3)	1 (reference)	-	-
**Aware of healthcare campaigns relating to Buruli ulcer**					
Yes	43 (48.9)	107(60.8)	0.5	0.29–0.97	0.040
No	45 (51.1)	69(39.2)	1(reference)	-	-
**Precautions taken against Buruli ulcer**					
Yes	16 (18.2)	56 (31.8)	0.4	0.23–0.86	0.016
No	72 (81.8)	120 (68.2)	1 (reference)	-	

**Note:** OR, unadjusted odds ratio for the comparison of cases with their corresponding controls; CI, confidence interval.

Multivariable analysis highlighted “lowland site” as a variable associated with a high risk of Buruli ulcer (Table 5). In our multivariable model, the following risk factors were independently associated with Buruli ulcer: principal source/body of water used located in a lowland area (OR = 10.8; 95% CI = 1.09–107.59), fetching water weekly from outdoors (OR = 3.9; 95% CI = 1.30–11.73), washing outside several times per day (OR = 10.4; 95% CI = 3.29–32.61), washing clothes unaccompanied (OR = 3.3; 95% CI = 1.28–8.48), washing the dishes occasionally (OR = 6.2; 95% CI = 1.43–27.23), and washing the dishes weekly (OR = 4.3; 95% CI = 1.03–18.21). By contrast, knowledge about Buruli ulcer (OR = 0.2; 95% CI = 0.07–0.39) was a protective factor.

**Table 5:** Multivariable analysis of the activities involving contact with water of the BU cases and controls matched for sex and age (± 1 year) living along the river Ouémé, Benin, 2018–20.

Variables	Adjusted OR (95% CI)	*p-*value
Main water point (lowland)	10.8 (1.09–107.59)	0.042
Fetching water outside weekly	3.9 (1.30–11.73)	0.015
Washing outside several times per day	10.4 (3.29–32.61)	0.000
Washing clothes alone	3.3 (1.28–8.48)	0.013
Washing the dishes occasionally	6.2 (1.43–27.23)	0.015
Washing the dishes weekly	4.3 (1.03–18.21)	0.046
Knowledge about Buruli ulcer	0.2 (0.07–0.39)	0.000

**Note:** OR, unadjusted odds ratio for the comparison of cases with the corresponding controls; CI, confidence interval.

### 3. Lowland location as a high-risk factor

#### 3.1 Distance to an area under water and the number of Buruli ulcer cases

We conducted a spatial analysis to investigate the “lowland” variable identified as associated with a high risk of Buruli ulcer contamination in more detail. On monthly radar images, we were able to distinguish an area permanently under water corresponding to the Ouémé River and a seasonally flooded area (in blue and yellow, respectively, in Fig 3D). As the radar images could only detect bodies of open water, we also used optical images (Sentinel 2 and Spot 5) to identifiy areas under humid forest or vegetation that were seasonally flooded (in pink in Fig 3D). We then estimated the distance between the location of the patient’s home and these bodies of water (permanent and/or seasonal). The number of Buruli ulcer cases increased with proximity to an area of water, from 10 cases at 1200 m to 70 cases at less than 400 m (Fig 3E). The increase was much more pronounced for semi-permanent or seasonal sites (from 0 cases at 1200 m to 53 cases at less than 400 m) than for the permanent surface waters corresponding to the Ouémé river (from 8 cases at 1200 m to 19 cases at less than 400 m) (Fig 3E).

#### 3.2 Environmental tracking of *M*. *ulcerans* DNA

**Fig 4:**
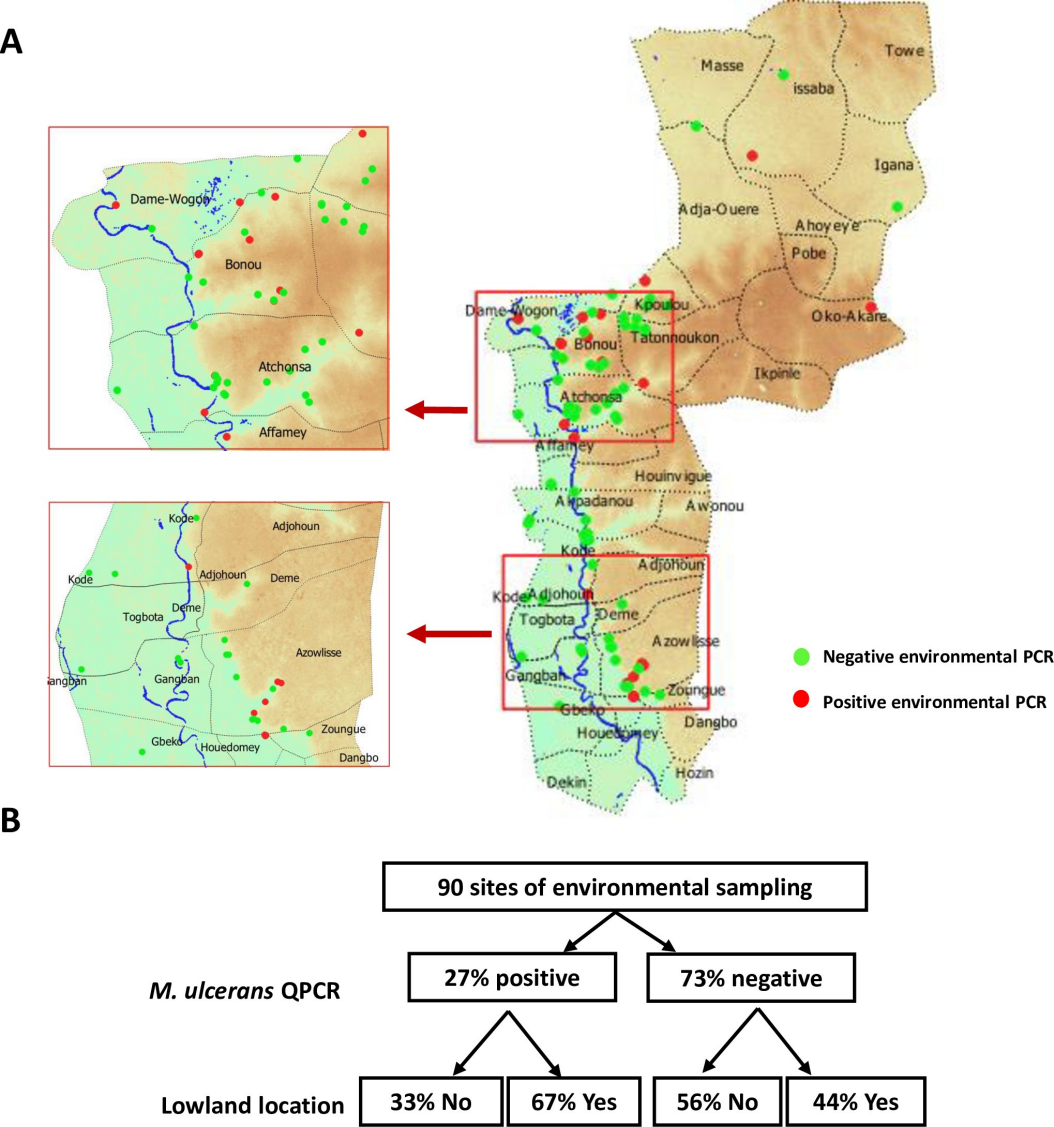
Detection of *M*. *ulcerans* at water sites. (A) Location of water sites from which environmental samples were collected. In green, water sites at which none of the pooled samples was positive for *M*. *ulcerans* DNA, and, in red, water sites at which at least one pooled sample tested positive for *M*. *ulcerans* DNA. Datas for the altitude and land surface are available on https://earthexplorer.usgs.gov/ (NASA), and https://apps.sentinel-hub.com/eo-browser/ (European Spatial Agency). Datas for the administrative boundary were provided by Ministry of Territory Planning of Benin (https://gadm.org/download_country_v3.html). (B) Proportion of positive water sites located in lowlands.

During the case-control study, we sampled the principal and/or secondary natural sources/bodies of water used by the participants. We looked for *M*. *ulcerans* DNA in aquatic animals and plants at 90 sampling sites. The plants and animals sampled were identified to order and family levels. In total, 55 of the 815 pools sorted by families were positive for *M*. *ulcerans* DNA (IS2404 and KR < 36 ct and 2 ct of difference) (S2 Table and S1 Fig). We found that 24 sites (27%) had at least one positive pool (Fig 4A and 4B). Overall, 67% of the sites testing positive for *M*. *ulcerans* DNA were in lowland areas, whereas only 42% of the negative sites were in lowland areas (*p* = 0.025, Fig 4B). Most of the positive sites were located at the border between wetlands and non-wetlands (Fig 4A).

#### 3.3 Link between variation in the area under water and endemicity

**Fig 5:**
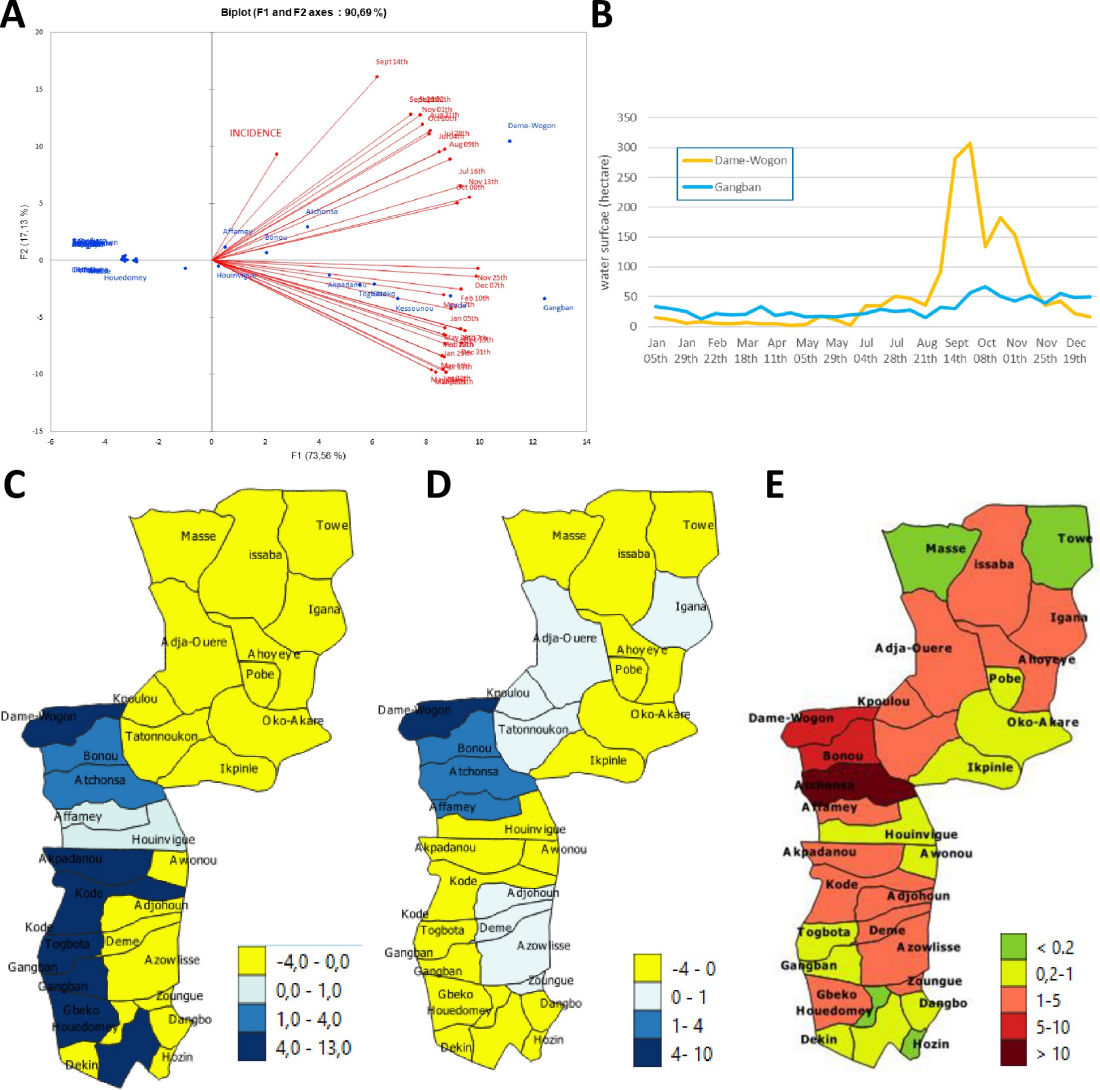
Link between the area under water and endemicity. (A) PCA analysis with monthly data for water coverage by district and endemicity. (B) Example of surface water area for two districts: Gangban and Dame-Wogon. (C) Projection of axis 1 of the PCA. (D) projection of axis 2 of the PCA. (E) Incidence of Buruli ulcer in the districts of the study area. Datas for the administrative boundary were provided by Ministry of Territory Planning of Benin (https://gadm.org/download_country_v3.html).

We also performed monthly analyses of the area under water in each district of our study area (11 in Plateau and 20 in Ouémé). We ran a PCA with incidence and monthly area under water as variables. Axis 1 (73% of the total variance) was positively correlated with all monthly water area variables regardless of season (Fig 5A), and corresponds to the water area distinguishing between the districts crossed by the Ouémé River and the dry districts located on the plateau (Fig 5C). Interestingly, axis 2 (17% of the total variance) provided additional information (Fig 5A and 5D): this axis was positively correlated with the wet months from July to November and inversely correlated with the other months. It distinguished between districts in which the area under water varied little between months (Gangban, Fig 5A, 5B and 5D) and those with a high degree of variation for the area under water (Dame-Wogon, Fig 5A, 5B and 5D). One key finding was the positive correlation between Buruli ulcer incidence and axis 2, indicating a higher risk of Buruli ulcer in districts with high degrees of seasonal variation for the area under water. These highly seasonal districts (Fig 5D) had more than one new case each year (Fig 5E), whereas districts without such seasonality in the area under water had smaller numbers of cases, even if crossed by the Ouémé river. This situation is illustrated by the contrast (Fig 5B) between the Dame-Wogon district, which is marked by a large seasonal variation of the area under water (Fig 5B) and the occurrence of more than five cases per year, and Gangban, which has a stable area permanently under water and less than one case per year (Fig 5E).

## Discussion

Research has provided little information to date, at individual level, about the reasons for which one person becomes contaminated whereas another does not, or at communal level, concerning the modes of community management that could potentially protect against disease or accentuate disease risk. Most of the case-control studies performed to date took place in hospitals or health centres, and few, if any, studies have been performed in the place of residence of the patients. These data collection methods contribute to a lack of precision concerning interactions with local aquatic environments and real behaviour, and have limited detailed epidemiological explorations. The limitations of this approach have been reported in several studies conducted in West Africa [46, 47]. We performed a combined analysis of epidemiological, geographic and spatial data, together with *M*. *ulcerans* detection over the same period, to increase our knowledge of the risk of infection and the factors protecting against it. This is the first detailed study, to our knowledge, to have applied such multidisciplinary approaches simultaneously during the same period.

Our pilot health geography study identified two activities (collecting water and crossing a water course) that accounted for more than 80% of all activities observed, and were predominantly performed by adult women. As previously reported [12], we identified a clear distortion of the sex ratio in adult Buruli ulcer patients in this area, with higher rates of *M*. *ulcerans* infection in adult women than in adult men. This led us to hypothesise that these activities are associated with a higher risk of infection. The variable “collecting water” (for drinking, cooking, washing up or washing at home) was identified as a risk factor both in this prospective cohort and in other case-control studies performed in Benin and published between 2005 and 2010 [47, 48]. The important role played by women in this activity was supported by data from our field observations and other social science studies [49, 50]. However, the risk of contamination can be considered low if the water source visited is flowing rather than stagnant, the surrounding areas are regularly maintained and if the site is organised so as to maximise protections [51].

The activity “crossing a water course” on foot to reach cultivated fields or a neighbouring village is a risk factor recorded in more recent case-control studies, particularly in Ghana and Togo [10, 13, 52], probably because this practice can be observed only if the observer remains in place for a long period. This activity results in prolonged contact with water, in turn promoting contact with *M*. *ulcerans* through contact with aquatic plants or animals, particularly during the rainy season. Prolonged walking in water was most frequently observed at the perimeter of the lowlands. Published studies have shown that the extent of lesions is significantly correlated with the area of the body left exposed [9]. More than 60% of lesions are found on the lower parts of the body (mostly on the legs). We saw young children remove most of their clothes before crossing, whereas adult women rolled up the lower part of their garments, thereby exposing the lower part of the bodies.

During field observations, “swimming” was a much less frequent activity than “collecting water” and “crossing a water course”, but we also suspect that this practice and the behaviours associated with it are risk factors for contamination. Children under the age of 15 years account for more than 50% of Buruli ulcer patients. Some studies have reported similar distributions of cases between boys and girls [46], but others have reported a higher prevalence in young boys than in girls [12, 48]. Our field observations suggest that swimming or wading in rivers is the only activity involving only children, and boys in particular, without the protection of clothing, particularly in stagnant water during the rainy season, which increases the risk of exposure.

One of the key strengths of our case-control study is the recruitment of incident cases, facilitating a detailed assessment of exposure over a well-defined period, during the six months before symptom onset. Case-control studies provide a robust design, but information and selection biases may nevertheless occur. The questionnaires were administered shortly after patient diagnosis. Controls were matched to cases to ensure comparability for potential confounding factors. One of the limitations of this type of study lies in the choice of control group and the quality of exposure measurement. However, the adjustment for confounding factors and multivariable analysis made it possible to minimise bias. Furthermore, the results of this case-control study are consistent with the socio-environmental data reported here, confirming our findings.

In a previous study, we found a correlation between the increase in the number of new wells and the decrease in the incidence of Buruli ulcer in some villages of northern Ouémé in which this disease is endemic [3]. In our prospective case-control study, we found that the use of protected sources of clean water (water from drilled boreholes, wells) was more frequently reported by controls than by cases. We also found that being the sole person responsible for fetching water from outside and large households were independently associated with Buruli ulcer incidence. The relationship between the larger numbers of people per household and Buruli ulcer could potentially be explained by larger households requiring more water, creating a greater risk of contact with contaminated water at unprotected water sources. We found that it was not only contact with unprotected natural water sources that was most associated with risk, but also the frequency of such contact, particularly visiting such sources alone, several times per day, particularly for water resources located in regularly flooded or irrigated lowlands.

In parallel, a high prevalence of Buruli ulcer was recorded in the villages close to lowland sources/bodies of water. *M*. *ulcerans* was more frequently detected in aquatic organisms sampled from the floodplain of the Ouémé River than from water sources at higher altitudes. The radar images highlighted a closer link between Buruli ulcer incidence and the temporary submersion of land by flood and runoff waters than between this incidence and permanent areas under water. In these districts, the area under water and the depth of the water in aquatic areas fluctuate considerably between seasons. These results support our previous scenarios suggested that, once introduced into a new environment, *M*. *ulcerans* expands rather than becoming a quiescent pathogen [53]. They also suggest that *M*. *ulcerans* can be introduced at sites temporarily or semi-permanently under water, subsequently disappearing, as in the villages of endemic Buruli ulcer located in Plateau.

The first step towards the planning of a preventive strategy in the study area is the identification, in each village, of specific unprotected water sources representing a risk for *M*. *ulcerans* contamination. Potential transmission sites meet at least one of the following criteria: (i) stagnant surface water or slow-flowing watercourses, or (ii) a seasonally flooded site after the rainy season. Permanent water sources outside lowland areas and water sources characterised by flowing water or regular maintenance of the surrounding area are not considered to be at risk. At the selected sites, the target population for preventive action consists of the children and adults using the source/body of water. A simple and clear preventive message could be delivered: (i) use water from wells or drilled boreholes for all domestic needs, (ii) avoid contact with water from unprotected and natural water sources, particularly during flooding, and (iii) avoid crossing or walking through water without protective clothing, particularly over long distances. At the scale of the village or community, prevention can be based around the following actions: (i) drilling to provide access to clean water throughout the year, (ii) building bridges and earthen levees to enable communities to move around without having to walk in the water, or promote the use of inland waterway boats to limiting walking in water during the wet season, and (iii) promoting the use of free surface water from non-lowland sites for domestic activities.

Our results also demonstrate the benefits of flexibility, reasoning not according to specific sites, but according to places of residence including both “safe” points of access to water and other points of access to water, at which humans may be exposed to *M*. *ulcerans*. Some of these unsafe points of access to water may be located in potentially risky semi-permanent or seasonal wetlands visited for reasons other than domestic activities. Indeed, behaviours and territorial practices in the lowland areas are far from limited to the activities described in this work. As a means of diversifying their income and supplying the markets of the towns and their surrounding areas with fresh produce, the communities have developed rice and maize production and market gardening activities [54]. Since the 1980s, runoff and irrigation networks have been traced out, to improve the use of the damp and fertile low-lying areas. One possible consequence of these hydroagricultural developments is the modification of natural habitats, favouring the development of *M*. *ulcerans*, certain vectors, or certain ecological reservoirs. These hypotheses have also received support from other studies performed in Benin [55, 56] and highlighting a progressive decrease in incidence with the development and clearing of low-lying areas and improvements in water management techniques. In our prospective cohort study, we detected the presence of *M*. *ulcerans* in aquatic organisms collected from fields flooded during a part of the year and frequented by some of the patients for agricultural work.

To our knowledge, no social science study has yet explored the question of agricultural behaviour and territorial practices in lowlands with a view to designing an appropriate preventive strategy. We still know little about these hydro-agricultural practices in low-lying areas, but they were shown to be associated with risk factors for exposure in recent case-control studies [9, 13, 24, 57] and they have not been analysed in a systematic manner. However, some information on agricultural work (soil preparation, planting, maintenance, and harvesting) indicates that most of the work is still done manually [55] and involves frequent contact with water. According to INSAE (the National Institute of Statistics and Economic Analysis) data, adult women make up 60% to 80% of the agricultural labour force in rural areas. However, the distribution of tasks between family members is not well documented and the role of women and children is underestimated [58]. The observation and characterisation of hydro-agricultural practices in the low-lying areas and of the organisation of labour represent a challenge in the definition of appropriate and effective preventive measures to reduce the incidence of infectious diseases related to water.

## Supporting information

S1 Table Types of water sources/bodies of water and Buruli ulcer incidence in 34 surveyed villages of the Plateau and Ouémé districts. (DOCX)

S2 Table Number of environmental samples collected in the study and detection of *M*. *ulcerans* by qPCR. (DOCX)

S1 Fig Number of pooled environmental samples testing positive for *M*. *ulcerans* by qPCR, by type of environmental sample. (DOCX)

S1 Data Questionnaire of the case-control study (English version). (DOCX)

S2 Data Questionnaire of the case-control study (French version). (DOCX)
