# Supplementary material for: A combined field study of Buruli ulcer disease in southeast Benin proposing preventive strategies based on epidemiological, geographic, behavioural and environmental analyses
Source: PLOS Glob Public Health. 2022 Jan 7;2(1):e0000095. doi: 10.1371/journal.pgph.0000095 (PMC10021984; doi:10.1371/journal.pgph.0000095)
Supplement: S1 Table — (DOCX) [file pgph.0000095.s001.docx]

**Table S1: Types of water sources/bodies of water and Buruli ulcer incidence in 34 surveyed villages of the Plateau and Ouémé districts**

| **Village** | **District** | **Incidence rate** | **Number of unprotected**  **water sources** | **Number of water sources in a lowland area (%)** | **River Ouémé** | **Tributary** | **Stream** | **Spring** | **Natural excavated area** | **Pond or water hole** |
| --- | --- | --- | --- | --- | --- | --- | --- | --- | --- | --- |
| Illemon | Plateau | 0 | 5 | 0 |  | 5 |  |  |  |  |
| Obeke-Ouere | Ouémé | 0.03 | 1 | 0 |  |  | 1 |  |  |  |
| Illoulofin | Plateau | 0.05 | 6 | 0 |  | 3 | 1 | 1 | 1 |  |
| Ketty | Plateau | 0.07 | 3 | 0 |  | 1 | 1 |  |  | 1 |
| Kpanoukpade | Ouémé | 0.07 | 1 | 1 (100%) |  |  |  |  | 1 |  |
| Trobossi | Ouémé | 0.13 | 4 | 0 |  | 1 | 2 | 1 |  |  |
| Kpoulou | Ouémé | 0.15 | 4 | 2 (50%) |  | 1 |  |  | 3 |  |
| Itchede | Plateau | 0.19 | 3 | 0 |  |  |  |  |  | 3 |
| Houezonme-Kpevi | Ouémé | 0.19 | 2 | 2(100%) |  |  |  |  | 2 |  |
| Fingninkanme | Ouémé | 0.2 | 1 | 0 |  |  |  | 1 |  |  |
| Itchoche | Plateau | 0.22 | 1 | 0 |  |  |  |  |  | 1 |
| Issaba | Plateau | 0.29 | 10 | 0 |  |  | 2 |  |  | 8 |
| Onigbolo | Plateau | 0.29 | 3 | 0 |  | 1 |  | 1 |  | 1 |
| Houedame | Ouémé | 0.42 | 8 | 0 |  |  | 1 | 7 |  |  |
| Zoungue | Ouémé | 0.43 | 10 | 10 (100%) |  |  | 3 | 6 |  | 1 |
| Wovime | Ouémé | 0.45 | 2 | 1 (50%) | 1 |  |  |  | 1 |  |
| Ouebossou | Ouémé | 0.49 | 12 | 4 (25%) | 6 | 1 |  | 3 |  | 2 |
| Towi | Ouémé | 0.5 | 7 | 0 |  |  | 4 | 3 |  |  |
| Zoukou | Ouémé | 0.54 | 1 | 1 (100%) |  |  |  | 1 |  |  |
| Deme | Ouémé | 0.57 | 6 | 0 | 6 |  |  |  |  |  |
| Mitro | Ouémé | 0.64 | 3 | 3 (100%) |  |  | 3 |  |  |  |
| Sota | Ouémé | 0.65 | 3 | 3 (100%) |  |  |  | 2 |  | 1 |
| Agbosso | Ouémé | 0.66 | 1 | 1 (100%) |  |  |  | 1 |  |  |
| Yokon | Ouémé | 0.83 | 9 | 9 (100%) |  |  | 4 | 5 |  |  |
| Gbekandji I | Ouémé | 0.88 | 1 | 1 (100%) |  |  |  |  | 1 |  |
| Agbonan | Ouémé | 0.98 | 16 | 1 (6%) |  | 2 | 9 | 4 |  | 1 |
| Eguelou | Plateau | 0.98 | 4 | 2 (50%) |  | 2 |  |  | 1 | 1 |
| Affame Centre | Ouémé | 1.11 | 2 | 1 (50%) |  |  |  | 2 |  |  |
| Sissekpa | Ouémé | 1.68 | 18 | 11 (60%) |  | 4 | 2 | 12 |  |  |
| Agbomahan | Ouémé | 1.7 | 19 | 1 (5%) |  | 2 | 9 | 8 |  |  |
| Houeda | Ouémé | 2.24 | 7 | 7 (100%) |  |  | 6 | 1 |  |  |
| Dasso | Ouémé | 2.36 | 3 | 3 (100%) |  |  |  | 3 |  |  |
| Tode | Ouémé | 2.47 | 4 | 4 (100%) |  |  |  | 4 |  |  |
| Olohougbodje | Ouémé | 4.07 | 9 | 4 (44%) |  | 5 | 4 |  |  |  |
