# Supplementary material for: A combined field study of Buruli ulcer disease in southeast Benin proposing preventive strategies based on epidemiological, geographic, behavioural and environmental analyses
Source: PLOS Glob Public Health. 2022 Jan 7;2(1):e0000095. doi: 10.1371/journal.pgph.0000095 (PMC10021984; doi:10.1371/journal.pgph.0000095)
Supplement: S2 Table — (DOCX) [file pgph.0000095.s002.docx]

**Table S2 : Number of environmental samples collected in the study and detection of *M. ulcerans* by qPCR**

| **Type of samples** | **Number of pooled samples** | **Number of qPCR-positive pooled samples*** | **Number of qPCR-negative pooled samples** | **% positive pooled samples** |
| --- | --- | --- | --- | --- |
| AQUATIC PLANTS | 292 | 6 | 286 | 2% |
| ORGANIC MATERIAL | 85 | 1 | 84 | 1% |
| VERTEBRATES** | 66 | 2 | 64 | 3% |
| COLEOPTERA | 66 | 4 | 62 | 6% |
| HETEROPTERA | 222 | 29 | 193 | 13% |
| ORTHOPTERA | 9 | 3 | 6 | 33% |
| HYMENOPTERA | 11 | 2 | 9 | 18% |
| OTHER INVERTEBRATES*** | 64 | 8 | 56 | 13% |
| **Total** | **815** | **55** | **760** | 7% |

***** Samples were considered positive only if both the IS*2404* sequence and the sequence encoding the KR domain of *mls* were detected, with threshold cycle (Ct) values strictly <36 cycles.

** Vertebrates include fish and amphibians

***Other invertebrates include molluscs, arachnids, decapods and dragonflies.
