# Supplementary material for: A combined field study of Buruli ulcer disease in southeast Benin proposing preventive strategies based on epidemiological, geographic, behavioural and environmental analyses
Source: PLOS Glob Public Health. 2022 Jan 7;2(1):e0000095. doi: 10.1371/journal.pgph.0000095 (PMC10021984; doi:10.1371/journal.pgph.0000095)
Supplement: S1 Fig — (DOCX) [file pgph.0000095.s003.docx]

**Figure S1: Number of pooled environmental samples testing positive for *M. ulcerans* by qPCR, by type of environmental sample**

**
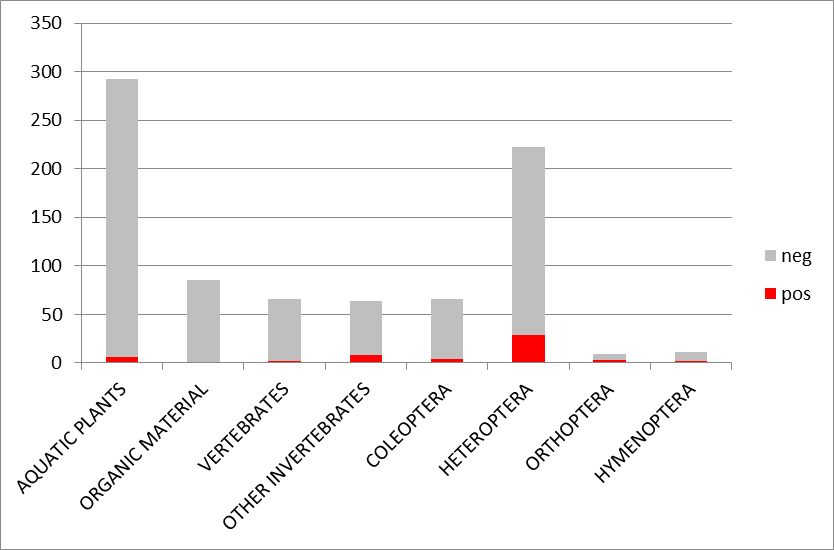
**
