# Supplementary material for: A combined field study of Buruli ulcer disease in southeast Benin proposing preventive strategies based on epidemiological, geographic, behavioural and environmental analyses
Source: PLOS Glob Public Health. 2022 Jan 7;2(1):e0000095. doi: 10.1371/journal.pgph.0000095 (PMC10021984; doi:10.1371/journal.pgph.0000095)
Supplement: S1 Data — (DOCX) [file pgph.0000095.s004.docx]

| **S1 Data: Questionnaire of the case-control study (English version)**  ***GéAnt*** |
| --- |

| \| **1. Date** \| \| --- \| |
| --- | --- |
|  |

| \| **2. Subject code (000-H/F-0/1/2)** \| \| --- \| |
| --- | --- |
|  |

| \| **3. LAST AND FIRST NAMES OF THE INTERVIEWER** \| \| --- \| |
| --- | --- |
|  |

| \| **IDENTITY OF THE PATIENT/CONTROL** \| \| --- \| |
| --- | --- |

| \| **4. Last Name** \| \| --- \| |
| --- | --- |
|  |

| \| **5. First Name** \| \| --- \| |
| --- | --- |
|  |

| \| **6. SEX** \| \| --- \| |
| --- | --- |
| \| 🔾 M \| 🔾 F \| \| --- \| --- \| |

| \| **7. Date of Birth** \| \| --- \| |
| --- | --- |
|  |

| \| **8-12. Place of residence (*département* (county), town, district, village, quarter/house)** \| \| --- \| |
| --- | --- |
| \| *Département* (county) \|  \| \| --- \| --- \| \| Town \|  \| \| District \|  \| \| Village \|  \| \| Quarter/house \|  \| |

| \| **13. GPS coordinates of the place of residence** \| \| --- \| |
| --- | --- |
|  |

| \| **14. How long have you been living in this village/quarter (in years)?** \| \| --- \| |
| --- | --- |
|  |

| \| **15. Ethnic group** \| \| --- \| |
| --- | --- |
|  |

| \| **16. Religion** \| \| --- \| |
| --- | --- |
|  |

| ***GéAnt*** |
| --- |

| \| **17-24. Current occupation** \| \| --- \| |
| --- | --- |
| \| Principal occupation \|  \| \| --- \| --- \| \| Since when? (P1) \|  \| \| AND where? (P1) \|  \| \| GPS1 \|  \| \| Secondary occupations \|  \| \| Since when? (P2) \|  \| \| And where? (P2) \|  \| \| GPS2 \|  \| |

| \| **25. How many people are there in your household (including yourself)?** \| \| --- \| |
| --- | --- |
|  |

| \| **26. Description of the household** \| \| --- \| |
| --- | --- |
|  |

| ***GéAnt*** |
| --- |

| \| **Everyday activities** \| \| --- \| |
| --- | --- |

| \| **27. During the week. Describe your principal activities on a usual day, specifying the times and places. Note for the interviewer: Write everything down in detail to make it easier to understand the subject’s lifestyle. Do not forget to note meals, the times at which the subject gets up and goes to bed, school, modes of transport, etc.** \| \| --- \| |
| --- | --- |
|  |

| \| **28. During the weekend. Describe your principal activities on a usual day, specifying the times and places. Note for the interviewer: Write everything down in detail to make it easier to understand the subject’s lifestyle. Do not forget to note meals, the times at which the subject gets up and goes to bed, school, modes of transport, etc.** \| \| --- \| |
| --- | --- |
|  |

| ***GéAnt*** |
| --- |

| \| **29-38. Which of the following activities are your responsibility for your household?** \| \| --- \| |
| --- | --- |
| \|  \| Always \| Sometimes \| Never \| Seasonal \| \| --- \| --- \| --- \| --- \| --- \| \| Cooking \| 🔾 \| 🔾 \| 🔾 \| 🔾 \| \| Washing dishes at home \| 🔾 \| 🔾 \| 🔾 \| 🔾 \| \| Laundry at home \| 🔾 \| 🔾 \| 🔾 \| 🔾 \| \| Cleaning the house \| 🔾 \| 🔾 \| 🔾 \| 🔾 \| \| Fetching water \| 🔾 \| 🔾 \| 🔾 \| 🔾 \| \| Washing motorcycles/other vehicles \| 🔾 \| 🔾 \| 🔾 \| 🔾 \| \| Shopping \| 🔾 \| 🔾 \| 🔾 \| 🔾 \| \| Hunting \| 🔾 \| 🔾 \| 🔾 \| 🔾 \| \| Fishing \| 🔾 \| 🔾 \| 🔾 \| 🔾 \| \| Kitchen gardening \| 🔾 \| 🔾 \| 🔾 \| 🔾 \| |

| \| **39. Explain why some of these activities are seasonal** \| \| --- \| |
| --- | --- |
|  |

| \| **40. During the last year, have you traveled anywhere?** \| \| --- \| |
| --- | --- |
| \| 🔾 Yes \| 🔾 No \| \| --- \| --- \| |

| \| **41-44. If yes,** \| \| --- \| |
| --- | --- |
| \| When? \|  \| \| --- \| --- \| \| For how long? \|  \| \| Where? \|  \| \| Why? \|  \| |

| \| **Water use** \| \| --- \| |
| --- | --- |

| \| **45. Do you have a source of water at your home?** \| \| --- \| |
| --- | --- |
| \| ❑ No \| ❑ Borehole \| \| --- \| --- \| \| ❑ Water tank \| ❑ Other \| \| ❑ Sink \|  \|  \| If 'Other' please specify:  \| \| --- \| |

| \| **47. Do you use it?** \| \| --- \| |
| --- | --- |
| \| 🔾 Yes \| 🔾 No \| \| --- \| --- \| |

| ***GéAnt*** |
| --- |

| \| **48-55. Which of the following 8 water-related activities do you engage in?** \| \| --- \| |
| --- | --- |
| \|  \| Several times per day \| Daily \| Weekly \| Occasionally \| No \| Early in the morning (before 7 a.m) \| Between 7 a.m and 12 noon \| Between 12 noon and 5 p.m. \| After 5 p.m. \| Entering a body of water \| Without entering a body of water \| Wearing sandals \| Wearing closed footwear \| Wearing non-closed footwear \| Wearing long clothes \| \| --- \| --- \| --- \| --- \| --- \| --- \| --- \| --- \| --- \| --- \| --- \| --- \| --- \| --- \| --- \| --- \| \| Fetching water from outside \| 🔾 \| 🔾 \| 🔾 \| 🔾 \| 🔾 \| 🔾 \| 🔾 \| 🔾 \| 🔾 \| 🔾 \| 🔾 \| 🔾 \| 🔾 \| 🔾 \| 🔾 \| \| Washing outside \| 🔾 \| 🔾 \| 🔾 \| 🔾 \| 🔾 \| 🔾 \| 🔾 \| 🔾 \| 🔾 \| 🔾 \| 🔾 \| 🔾 \| 🔾 \| 🔾 \| 🔾 \| \| Swimming \| 🔾 \| 🔾 \| 🔾 \| 🔾 \| 🔾 \| 🔾 \| 🔾 \| 🔾 \| 🔾 \| 🔾 \| 🔾 \| 🔾 \| 🔾 \| 🔾 \| 🔾 \| \| Laundry outside \| 🔾 \| 🔾 \| 🔾 \| 🔾 \| 🔾 \| 🔾 \| 🔾 \| 🔾 \| 🔾 \| 🔾 \| 🔾 \| 🔾 \| 🔾 \| 🔾 \| 🔾 \| \| Washing the dishes outside \| 🔾 \| 🔾 \| 🔾 \| 🔾 \| 🔾 \| 🔾 \| 🔾 \| 🔾 \| 🔾 \| 🔾 \| 🔾 \| 🔾 \| 🔾 \| 🔾 \| 🔾 \| \| Contact with water during professional activities. \| 🔾 \| 🔾 \| 🔾 \| 🔾 \| 🔾 \| 🔾 \| 🔾 \| 🔾 \| 🔾 \| 🔾 \| 🔾 \| 🔾 \| 🔾 \| 🔾 \| 🔾 \| \| Fishing \| 🔾 \| 🔾 \| 🔾 \| 🔾 \| 🔾 \| 🔾 \| 🔾 \| 🔾 \| 🔾 \| 🔾 \| 🔾 \| 🔾 \| 🔾 \| 🔾 \| 🔾 \| \| Other \| 🔾 \| 🔾 \| 🔾 \| 🔾 \| 🔾 \| 🔾 \| 🔾 \| 🔾 \| 🔾 \| 🔾 \| 🔾 \| 🔾 \| 🔾 \| 🔾 \| 🔾 \| |

| \| **56. If you come into contact with water during your professional activities, please explain how:** \| \| --- \| |
| --- | --- |
|  |

| \| **57. Other types of contact with water (please provide details)** \| \| --- \| |
| --- | --- |
|  |

| \| **58-65. For each of these activities, where do you perform the activity? (type of site, e.g. river, spring etc., and, if possible, the name of the site)** \| \| --- \| |
| --- | --- |
| \| Fetching water from outside \|  \| \| --- \| --- \| \| Washing outside \|  \| \| Swimming \|  \| \| Laundry outside \|  \| \| Washing the dishes outside \|  \| \| Contact with water during professional activities \|  \| \| Fishing \|  \| \| Other \|  \| |

| ***GéAnt*** |
| --- |

| \| **66-73. For each of these activities, what are the GPS coordinates of the principal site?** \| \| --- \| |
| --- | --- |
| \| Fetching water from outside \|  \| \| --- \| --- \| \| Washing outside \|  \| \| Swimming \|  \| \| Laundry outside \|  \| \| Washing dishes outside \|  \| \| Contact with water during professional activities \|  \| \| Fishing \|  \| \| Other \|  \| |

| \| **74-81. For each of these activities, what are the GPS coordinates of the secondary site?** \| \| --- \| |
| --- | --- |
| \| Fetching water from outside \|  \| \| --- \| --- \| \| Washing outside \|  \| \| Swimming \|  \| \| Laundry outside \|  \| \| Washing dishes outside \|  \| \| Contact with water during professional activities \|  \| \| Fishing \|  \| \| Other \|  \| |

| \| **82-89. For each of these activities, do you perform the activity?** \| \| --- \| |
| --- | --- |
| \|  \| Alone \| Accompanied \| The person who accompanies me most frequently has contracted BU \| The person who accompanies me the most frequently has not contracted BU \| \| --- \| --- \| --- \| --- \| --- \| \| Fetching water from outside \| 🔾 \| 🔾 \| 🔾 \| 🔾 \| \| Washing outside \| 🔾 \| 🔾 \| 🔾 \| 🔾 \| \| Swimming \| 🔾 \| 🔾 \| 🔾 \| 🔾 \| \| Laundry outside \| 🔾 \| 🔾 \| 🔾 \| 🔾 \| \| Washing dishes outside \| 🔾 \| 🔾 \| 🔾 \| 🔾 \| \| Contact with water during professsional activities \| 🔾 \| 🔾 \| 🔾 \| 🔾 \| \| Fishing \| 🔾 \| 🔾 \| 🔾 \| 🔾 \| \| Other \| 🔾 \| 🔾 \| 🔾 \| 🔾 \| |

| \| **90. If you are often with a particular person, who is it?** \| \| --- \| |
| --- | --- |
|  |

| ***GéAnt*** |
| --- |

| \| **91. Free comment about contact with water. Description of the various sites, and of the organization of human activities at water sources. Description of changes during flooding.............. Changes in everyday practices during different seasons, holidays, etc. ............** \| \| --- \| |
| --- | --- |
|  |

| \| **Relationship with the illness (cases)** \| \| --- \| |
| --- | --- |

| \| **92. Do you remember when and how the first symptoms of the disease occurred?** \| \| --- \| |
| --- | --- |
| \| 🔾 Yes \| 🔾 No \| \| --- \| --- \| |

| \| **93. If yes, how?** \| \| --- \| |
| --- | --- |
| \| ❑ Plaque \| ❑ Swelling \| \| --- \| --- \| \| ❑ Nodule \| ❑ Spot \| \| ❑ Edema \|  \| |

| \| **94. Tell us about it:** \| \| --- \| |
| --- | --- |
|  |

| \| **95. How and where do you think you caught the disease? In your opinion, what are the causes of this disease? Tell us about it. Note for the interviewer: if a particular site is mentioned, you should go to this site, describe it and take GPS coordinates. Note: go back to the question about travel. If the person reports having traveled, do they think they could have contracted Buruli ulcer at that time?** \| \| --- \| |
| --- | --- |
|  |

| ***GéAnt*** |
| --- |

| \| **96. During the weeks preceding the appearance of the wound, did you feel any insect bites or scratches from plants during your everyday activities? Note to the interviewer: at the end of the subject’s account, ask about the frequency of such bites/scratches (rare or frequent?)** \| \| --- \| |
| --- | --- |
| \| 🔾 Yes \| 🔾 If yes, please provide more information \| \| --- \| --- \| \| 🔾 No \|  \|  \| If yes, tell us about it  \| \| --- \| |

| \| **98. During the weeks preceding the appearance of the wound, did you experience any injuries, collisions, itching or cuts during your everyday activities?** \| \| --- \| |
| --- | --- |
|  |

| \| **99. During the appearance of the wound, how did you treat it? Did you consult anyone before you were treated by the CDTLUB?** \| \| --- \| |
| --- | --- |
|  |

| \| **100. Did you know about Buruli ulcer before contracting the disease yourself?** \| \| --- \| |
| --- | --- |
| \| 🔾 Yes \| 🔾 No \| \| --- \| --- \| |

| \| **101. If yes, how did you know about it?** \| \| --- \| |
| --- | --- |
|  |

| \| **102. Have you heard or seen any Buruli ulcer awareness campaign messages?** \| \| --- \| |
| --- | --- |
| \| 🔾 Yes \| 🔾 No \| \| --- \| --- \| |

| \| **103. If yes, please specify where? (radio, school, health center, town criers)** \| \| --- \| |
| --- | --- |
| \| ❑ Radio \| ❑ Health center \| \| --- \| --- \| \| ❑ School \| ❑ Other \|  \| If 'Other' please specify:  \| \| --- \| |

| \| **105. Did you take any particular precautions to avoid catching the disease?** \| \| --- \| |
| --- | --- |
| \| 🔾 Yes \| 🔾 No \| \| --- \| --- \| |

| ***GéAnt*** |
| --- |

| \| **106. If yes, which measures did you take?** \| \| --- \| |
| --- | --- |
|  |

| \| **107. Do you know anyone among your family and friends who has had Buruli ulcer?** \| \| --- \| |
| --- | --- |
| \| 🔾 Yes \| 🔾 No \| \| --- \| --- \| |

| \| **108. If yes: please specify who and when (in which year?)** \| \| --- \| |
| --- | --- |
|  |

| \| **Relationship with the disease (controls)** \| \| --- \| |
| --- | --- |

| \| **109. Have you ever known anyone with Buruli ulcer before being asked to complete this questionnaire ? If the answer is ‘No’, please go to question 117.** \| \| --- \| |
| --- | --- |
| \| 🔾 Yes \| 🔾 No \| \| --- \| --- \| |

| \| **110. If yes, how did you know them?** \| \| --- \| |
| --- | --- |
|  |

| \| **111. Have you heard or seen any Buruli ulcer awareness campaign messages?** \| \| --- \| |
| --- | --- |
| \| 🔾 Yes \| 🔾 No \| \| --- \| --- \| |

| \| **112. If yes, where did they come from?** \| \| --- \| |
| --- | --- |
| \| ❑ Radio \| ❑ Town crier \| \| --- \| --- \| \| ❑ School \| ❑ Other \| \| ❑ Health center \|  \|  \| If 'Other' please specify:  \| \| --- \| |

| \| **114. Did you take any particular precautions to avoid catching the disease?** \| \| --- \| |
| --- | --- |
|  |

| \| **115. What do you think are the causes of this disease?** \| \| --- \| |
| --- | --- |
|  |
