# Supplementary material for: A combined field study of Buruli ulcer disease in southeast Benin proposing preventive strategies based on epidemiological, geographic, behavioural and environmental analyses
Source: PLOS Glob Public Health. 2022 Jan 7;2(1):e0000095. doi: 10.1371/journal.pgph.0000095 (PMC10021984; doi:10.1371/journal.pgph.0000095)
Supplement: S2 Data — (DOCX) [file pgph.0000095.s005.docx]

**S2 Data : Questionnaire of the case-control study (French version)**

| ***GéAnt*** |
| --- |

| \| **1. Date** \| \| --- \| |
| --- | --- |
|  |

| \| **2. Code Sujet (000-H/F-0/1/2)** \| \| --- \| |
| --- | --- |
|  |

| \| **3. NOM ET PRENOM DE L'ENQUETEUR** \| \| --- \| |
| --- | --- |
|  |

| \| **IDENTITE DU PATIENT/TEMOIN** \| \| --- \| |
| --- | --- |

| \| **4. Nom** \| \| --- \| |
| --- | --- |
|  |

| \| **5. Prénom** \| \| --- \| |
| --- | --- |
|  |

| \| **6. SEXE** \| \| --- \| |
| --- | --- |
| \| 🔾 M \| 🔾 F \| \| --- \| --- \| |

| \| **7. Date de naissance** \| \| --- \| |
| --- | --- |
|  |

| \| **8-12. Lieu de résidence(département, commune, arrondissement, village, quartier/maison)** \| \| --- \| |
| --- | --- |
| \| Département \|  \| \| --- \| --- \| \| Commune \|  \| \| Arrondissement \|  \| \| Village \|  \| \| Quartier/maison \|  \| |

| \| **13. Coordonnées GPS du lieu de résidence** \| \| --- \| |
| --- | --- |
|  |

| \| **14. Depuis quand vivez-vous dans le village/quartier (nombre d'années)** \| \| --- \| |
| --- | --- |
|  |

| \| **15. Origine ethnique** \| \| --- \| |
| --- | --- |
|  |

| \| **16. Confession religieuse** \| \| --- \| |
| --- | --- |
|  |

| ***GéAnt*** |
| --- |

| \| **17-24. Profession actuelle** \| \| --- \| |
| --- | --- |
| \| Principale \|  \| \| --- \| --- \| \| Depuis quand? (P1) \|  \| \| ET où ? (P1) \|  \| \| GPS1 \|  \| \| Professions secondaires \|  \| \| Depuis quand? (P2) \|  \| \| Et où ? (P2) \|  \| \| GPS2 \|  \| |

| \| **25. De combien de personnes se compose votre foyer (en vous incluant)** \| \| --- \| |
| --- | --- |
|  |

| \| **26. Description du foyer** \| \| --- \| |
| --- | --- |
|  |

| ***GéAnt*** |
| --- |

| \| **Habitudes de vie** \| \| --- \| |
| --- | --- |

| \| **27. En semaine. Décrivez-nous vos principales activités dans une journée habituelle en précisant les lieux et les heures? Consigne à l'enquêteur : Tout écrire avec détails pour comprendre son mode de vie. Ne pas oublier les repas, heure de levée, couché, école, mode de transport....** \| \| --- \| |
| --- | --- |
|  |

| \| **28. Le week-end. Décrivez-nous vos principales activités dans une journée habituelle en précisant les lieux et les heures? Consigne à l'enquêteur : Tout écrire avec détails pour comprendre son mode de vie. Ne pas oublier les repas, heure de levée, couché, école, mode de transport....** \| \| --- \| |
| --- | --- |
|  |

| ***GéAnt*** |
| --- |

| \| **29-38. Parmi les activités suivantes lesquelles sont à votre charge pour votre foyer?** \| \| --- \| |
| --- | --- |
| \|  \| Toujours \| Parfois \| Jamais \| Saisonnier \| \| --- \| --- \| --- \| --- \| --- \| \| Cuisine \| 🔾 \| 🔾 \| 🔾 \| 🔾 \| \| Vaisselle \| 🔾 \| 🔾 \| 🔾 \| 🔾 \| \| Lessive \| 🔾 \| 🔾 \| 🔾 \| 🔾 \| \| Ménage \| 🔾 \| 🔾 \| 🔾 \| 🔾 \| \| Aller chercher l'eau \| 🔾 \| 🔾 \| 🔾 \| 🔾 \| \| Laver les motos ou véhicules \| 🔾 \| 🔾 \| 🔾 \| 🔾 \| \| Faire les achats \| 🔾 \| 🔾 \| 🔾 \| 🔾 \| \| Chasser \| 🔾 \| 🔾 \| 🔾 \| 🔾 \| \| Pêcher \| 🔾 \| 🔾 \| 🔾 \| 🔾 \| \| Potager \| 🔾 \| 🔾 \| 🔾 \| 🔾 \| |

| \| **39. Précisez pourquoi pour certaines activités sont saisonnières** \| \| --- \| |
| --- | --- |
|  |

| \| **40. Au cours de l'année passée, avez-vous voyagé?** \| \| --- \| |
| --- | --- |
| \| 🔾 Oui \| 🔾 Non \| \| --- \| --- \| |

| \| **41-44. Si oui,** \| \| --- \| |
| --- | --- |
| \| Quand ? \|  \| \| --- \| --- \| \| Combien de temps ? \|  \| \| Où ? \|  \| \| Pour quel motif ? \|  \| |

| \| **Usages de l'eau** \| \| --- \| |
| --- | --- |

| \| **45. Avez-vous un point d'eau dans votre maison ?** \| \| --- \| |
| --- | --- |
| \| ❑ Non \| ❑ Forage \| \| --- \| --- \| \| ❑ Citerne \| ❑ Autre \| \| ❑ Lavabo \|  \|  \| Si 'Autre' précisez :  \| \| --- \| |

| \| **47. Est-ce que vous l'utilisez?** \| \| --- \| |
| --- | --- |
| \| 🔾 Oui \| 🔾 Non \| \| --- \| --- \| |

| ***GéAnt*** |
| --- |

| \| **48-55. Parmi les 8 activités suivantes en rapport avec l'eau, lesquelles pratiquez-vous ?** \| \| --- \| |
| --- | --- |
| \|  \| Plusieurs fois/jour \| Quotidien \| Hebdomadaire \| Occasionnel \| Non \| Tôt le matin (avant 7h) \| Entre 7 et 12 h \| Entre 12 et 17 h \| Après 17 h \| J'entre dans l'eau \| Je n'entre pas dans l'eau \| Je porte des chaussures ouvertes \| je porte des chaussures fermées \| Je ne porte pas de chaussures \| Je porte des vêtements longs \| \| --- \| --- \| --- \| --- \| --- \| --- \| --- \| --- \| --- \| --- \| --- \| --- \| --- \| --- \| --- \| --- \| \| Aller chercher de l'eau pour le foyer \| 🔾 \| 🔾 \| 🔾 \| 🔾 \| 🔾 \| 🔾 \| 🔾 \| 🔾 \| 🔾 \| 🔾 \| 🔾 \| 🔾 \| 🔾 \| 🔾 \| 🔾 \| \| Se laver à l'extérieur. A l'extérieur signifie, en dehors de la maison, dans une source par ex. \| 🔾 \| 🔾 \| 🔾 \| 🔾 \| 🔾 \| 🔾 \| 🔾 \| 🔾 \| 🔾 \| 🔾 \| 🔾 \| 🔾 \| 🔾 \| 🔾 \| 🔾 \| \| Se baigner à l'extérieur \| 🔾 \| 🔾 \| 🔾 \| 🔾 \| 🔾 \| 🔾 \| 🔾 \| 🔾 \| 🔾 \| 🔾 \| 🔾 \| 🔾 \| 🔾 \| 🔾 \| 🔾 \| \| Faire la lessive à l'extérieur \| 🔾 \| 🔾 \| 🔾 \| 🔾 \| 🔾 \| 🔾 \| 🔾 \| 🔾 \| 🔾 \| 🔾 \| 🔾 \| 🔾 \| 🔾 \| 🔾 \| 🔾 \| \| Faire la vaisselle à l'extérieur \| 🔾 \| 🔾 \| 🔾 \| 🔾 \| 🔾 \| 🔾 \| 🔾 \| 🔾 \| 🔾 \| 🔾 \| 🔾 \| 🔾 \| 🔾 \| 🔾 \| 🔾 \| \| Contact avec l'eau lors de de vos activités professionnelles. \| 🔾 \| 🔾 \| 🔾 \| 🔾 \| 🔾 \| 🔾 \| 🔾 \| 🔾 \| 🔾 \| 🔾 \| 🔾 \| 🔾 \| 🔾 \| 🔾 \| 🔾 \| \| Pêche loisirs \| 🔾 \| 🔾 \| 🔾 \| 🔾 \| 🔾 \| 🔾 \| 🔾 \| 🔾 \| 🔾 \| 🔾 \| 🔾 \| 🔾 \| 🔾 \| 🔾 \| 🔾 \| \| Autres \| 🔾 \| 🔾 \| 🔾 \| 🔾 \| 🔾 \| 🔾 \| 🔾 \| 🔾 \| 🔾 \| 🔾 \| 🔾 \| 🔾 \| 🔾 \| 🔾 \| 🔾 \| |

| \| **56. Si vous avez contact avec l'eau lors de vos activités professionnelles, précisez :** \| \| --- \| |
| --- | --- |
|  |

| \| **57. Autres contacts avec l'eau (développer)** \| \| --- \| |
| --- | --- |
|  |

| \| **58-65. Pour chacune de ces activités, où les faites vous ? (type de lieu ex. fleuve, source, ... + éventuellement nom du lieu)** \| \| --- \| |
| --- | --- |
| \| Aller chercher de l'eau pour le foyer \|  \| \| --- \| --- \| \| Se laver à l'extérieur. a l'extérieur signifie, en dehors de la maison, dans une source par ex. \|  \| \| Se baigner à l'extérieur \|  \| \| Faire la lessive à l'extérieur \|  \| \| Faire la vaisselle à l'extérieur \|  \| \| Contact avec l'eau lors d'activités professionnelles \|  \| \| Pêche loisirs \|  \| \| Autres \|  \| |

| ***GéAnt*** |
| --- |

| \| **66-73. Pour chacune de ces activités, quelles sont les coordonnées GPS du lieu principal ?** \| \| --- \| |
| --- | --- |
| \| Aller chercher de l'eau pour le foyer \|  \| \| --- \| --- \| \| Se laver à l'extérieur. a l'extérieur signifie, en dehors de la maison, dans une source par ex. \|  \| \| Se baigner à l'extérieur \|  \| \| Faire la lessive à l'extérieur \|  \| \| Faire la vaisselle à l'extérieur \|  \| \| Contact avec l'eau lors d'activités professionnelles \|  \| \| Pêche loisirs \|  \| \| Autres \|  \| |

| \| **74-81. Pour chacune de ces activités, coordonnées GPS du lieu secondaire ?** \| \| --- \| |
| --- | --- |
| \| Aller chercher de l'eau pour le foyer \|  \| \| --- \| --- \| \| Se laver à l'extérieur. a l'extérieur signifie, en dehors de la maison, dans une source par ex. \|  \| \| Se baigner à l'extérieur \|  \| \| Faire la lessive à l'extérieur \|  \| \| Faire la vaisselle à l'extérieur \|  \| \| Contact avec l'eau lors d'activités professionnelles \|  \| \| Pêche loisirs \|  \| \| Autres \|  \| |

| \| **82-89. Pour chacune de ces activités, les faites vous ?** \| \| --- \| |
| --- | --- |
| \|  \| Seul.e \| Accompagné.e \| La personne qui m'accompagne le plus souvent a contracté l'UB \| La personne qui m'accompagne le plus souvent n'a pas contracté l'UB \| \| --- \| --- \| --- \| --- \| --- \| \| Aller chercher de l'eau pour le foyer \| 🔾 \| 🔾 \| 🔾 \| 🔾 \| \| Se laver à l'extérieur. a l'extérieur signifie, en dehors de la maison, dans une source par ex. \| 🔾 \| 🔾 \| 🔾 \| 🔾 \| \| Se baigner à l'extérieur \| 🔾 \| 🔾 \| 🔾 \| 🔾 \| \| Faire la lessive à l'extérieur \| 🔾 \| 🔾 \| 🔾 \| 🔾 \| \| Faire la vaisselle à l'extérieur \| 🔾 \| 🔾 \| 🔾 \| 🔾 \| \| Contact avec l'eau lors d'activités professionnelles \| 🔾 \| 🔾 \| 🔾 \| 🔾 \| \| Pêche loisirs \| 🔾 \| 🔾 \| 🔾 \| 🔾 \| \| Autres \| 🔾 \| 🔾 \| 🔾 \| 🔾 \| |

| \| **90. Si vous êtes souvent avec quelqu'un, qui-est-ce?** \| \| --- \| |
| --- | --- |
|  |

| ***GéAnt*** |
| --- |

| \| **91. Commentaire libre de l'enquêteur sur contact avec l'eau : Description des différents sites, aménagement de l'Homme sur les sources. Description des changements lors de la crue.............. Changement des habitudes de vie pendant les différentes saisons, les congés...........** \| \| --- \| |
| --- | --- |
|  |

| \| **Rapport avec la maladie (cas)** \| \| --- \| |
| --- | --- |

| \| **92. Vous souvenez-vous de quand et comment sont apparus les premiers symptômes de la maladie ?** \| \| --- \| |
| --- | --- |
| \| 🔾 Oui \| 🔾 Non \| \| --- \| --- \| |

| \| **93. Si oui, comment ?** \| \| --- \| |
| --- | --- |
| \| ❑ Plaque \| ❑ Gonflement \| \| --- \| --- \| \| ❑ Nodule \| ❑ Bouton \| \| ❑ Oedème \|  \| |

| \| **94. Racontez-nous :** \| \| --- \| |
| --- | --- |
|  |

| \| **95. Comment et où pensez-vous avoir attrapé la maladie ? Selon vous, quelle(s) est (sont) les causes de cette maladie ? Racontez-nous ? Consigne à l'enquêteur : si un endroit est mentionné, se rendre sur le lieu, le décrire et prendre GPS. Attention, revenir à la question du voyage du patient. Si la personne a voyagé, pense t-il qu'il pourrait pu contracter l'Ulcère de Buruli à ce moment là ?** \| \| --- \| |
| --- | --- |
|  |

| ***GéAnt*** |
| --- |

| \| **96. Durant les semaines précédent l'apparition de la plaie, aviez-vous ressenti des piqûres d'insectes ou de plantes lors de vos activités journalières ? Consigne à l'enquêteur : à la fin du récit, demander la fréquence des piqûres (rare ou fréquent ?)** \| \| --- \| |
| --- | --- |
| \| 🔾 Oui \| 🔾 si oui racontez-nous \| \| --- \| --- \| \| 🔾 Non \|  \|  \| si oui racontez-nous  \| \| --- \| |

| \| **98. Durant les semaines précédant l'apparition de la plaie, aviez-vous eu des blessures, des chocs, des prurits, des coupures lors de vos activités journalières ?** \| \| --- \| |
| --- | --- |
|  |

| \| **99. Lors de l'apparition de la plaie, comment vous êtes-vous soigné ? Avez-vous consulté quelqu'un avant d'être pris en charge par le CDTLUB ?** \| \| --- \| |
| --- | --- |
|  |

| \| **100. Connaissiez-vous la maladie ulcère de Buruli avant d'être malade vous-même?** \| \| --- \| |
| --- | --- |
| \| 🔾 Oui \| 🔾 Non \| \| --- \| --- \| |

| \| **101. Si oui, comment l'avez-vous connu ?** \| \| --- \| |
| --- | --- |
|  |

| \| **102. Aviez-vous vous déjà entendu des messages de sensibilisation?** \| \| --- \| |
| --- | --- |
| \| 🔾 Oui \| 🔾 Non \| \| --- \| --- \| |

| \| **103. Si oui, précisez où ? (radio, école, centre de santé, crieurs publics)** \| \| --- \| |
| --- | --- |
| \| ❑ Radio \| ❑ Centre de santé \| \| --- \| --- \| \| ❑ Ecole \| ❑ Autre \|  \| Si 'Autre' précisez :  \| \| --- \| |

| \| **105. Aviez-vous pris des précautions particulières pour ne pas attraper la maladie?** \| \| --- \| |
| --- | --- |
| \| 🔾 Oui \| 🔾 Non \| \| --- \| --- \| |

| ***GéAnt*** |
| --- |

| \| **106. Si oui, lesquelles?** \| \| --- \| |
| --- | --- |
|  |

| \| **107. Connaissez-vous quelqu'un qui a fait l'ulcère de Buruli dans votre famille ou entourage?** \| \| --- \| |
| --- | --- |
| \| 🔾 Oui \| 🔾 Non \| \| --- \| --- \| |

| \| **108. Si oui : précisez qui et en qu'elle année?** \| \| --- \| |
| --- | --- |
|  |

| \| **Rapport avec la maladie (Témoins)** \| \| --- \| |
| --- | --- |

| \| **109. Connaissez-vous la maladie ulcère de Buruli avant ce questionnaire? Si la réponse est Non, passez à la question 117.** \| \| --- \| |
| --- | --- |
| \| 🔾 Oui \| 🔾 Non \| \| --- \| --- \| |

| \| **110. Si oui, comment l'avez-vous connu?** \| \| --- \| |
| --- | --- |
|  |

| \| **111. Avez-vous déjà entendu des messages de sensibilisation ?** \| \| --- \| |
| --- | --- |
| \| 🔾 Oui \| 🔾 Non \| \| --- \| --- \| |

| \| **112. Si oui, précisez par qui?** \| \| --- \| |
| --- | --- |
| \| ❑ Radios \| ❑ Crieur public \| \| --- \| --- \| \| ❑ Ecoles \| ❑ Autre \| \| ❑ Centres de santé \|  \|  \| Si 'Autre' précisez :  \| \| --- \| |

| \| **114. Avez-vous pris des précautions particulières pour ne pas attraper la maladie?** \| \| --- \| |
| --- | --- |
|  |

| \| **115. Selon vous, qu'elle(s) est(sont) les causes de cette maladie?** \| \| --- \| |
| --- | --- |
|  |
